# circRNA Signatures Distinguishing COVID-19 Outcomes and Acute Respiratory Distress Syndrome: A Longitudinal, Two-Timepoint, Precision-Weighted Analysis of a Public RNA-Seq Cohort

**DOI:** 10.3390/genes17010034

**Published:** 2025-12-30

**Authors:** Alawi Habara

**Affiliations:** Department of Biochemistry, College of Medicine, Imam Abdulrahman Bin Faisal University, P.O. Box 1982, Dammam 31441, Saudi Arabia; ahhabara@iau.edu.sa

**Keywords:** circular RNA, COVID-19, ARDS, longitudinal, differential expression, precision-weighted integration, RNA-seq, biomarker

## Abstract

Background: Although circular RNAs are increasingly implicated in host responses, their longitudinal behaviors to predict outcomes in severe COVID-19 remain unclear. The purpose of this study is to distinguish the circRNA signature associated with COVID-19 outcome. Method: Public total RNA-seq data from GEO (GSE273149) were used to assess circRNA differences among COVID-19 non-survivors, COVID-19 survivors, and patients with acute respiratory distress syndrome (ARDS) serving as severity-matched disease controls at two timepoints: Early (Day 3) and Late (Days 7 to 10). Differential expression was assessed after quality filtering, with the results reported as significant (FDR < 0.05) or suggestive (0.05–0.10); |log_2_FC| ≥ 1 was used as a guide for interpretation. Early and Late effects were combined using a two-timepoint, precision-weighted approach to prioritize time-consistent signals. Results: A distinction between non-survivors and survivors was observed, with nine significant and four suggestive candidates identified in the combined analysis; in addition, some candidates indicated a difference between survivors and ARDS controls. Early and Late effects primarily occurred in the same direction, and several circRNAs that were borderline at one timepoint became significant when the two timepoints were combined. Conclusion: This time-resolved, precision-weighted analysis of public RNA-seq data reveals stable circRNA differences between key clinical groups (patients with severe COVID-19 and those with ARDS), improving detection and interpretability relative to single-timepoint tests and yielding a concise set of candidates suitable for mechanistic follow-up and potential biomarker development.

## 1. Introduction

The causative agent of COVID-19 is severe acute respiratory syndrome coronavirus 2 (SARS-CoV-2), which has remained a significant global health burden since it was reported in late 2019 [1,2]. Although much has been accomplished in defining the behavior of viral transmission, immune responses, and clinical biomarkers, there is little information on the long-term responses of non-coding RNA species, particularly circular RNAs (circRNAs), during severe disease. circRNAs are covalently closed RNA species formed via back-splicing, and possess remarkable stability compared to linear RNAs [3,4]. Recent evidence suggests that viral and host circRNAs are involved in immune regulation, inflammatory signaling, and antiviral responses, and can serve as promising biomarker candidates [5,6].

However, most studies on circRNA in COVID-19 have been cross-sectional (Table 1), so they cannot identify time-dependent patterns or establish pathways of expression related to patients’ outcomes (survival vs. non-survival). Considering the dynamic immunological changes that take place in severe COVID-19, longitudinal profiling is essential to differentiate between temporary changes and long-term disease-wide modifications. Moreover, it can be more beneficial to combine information obtained at various timepoints with precision-weighted methods and enhance the discovery of resilient circRNA signatures that are not influenced by day-to-day biological changes [7,8].

To fill the gaps in the literature, this study re-examines a publicly available total RNA-seq dataset (GSE273149) derived from peripheral blood mononuclear cells (PBMCs) that were collected at two clinically relevant timepoints (Early: Day 3, and Late: Days 7–10). The aim was to determine circRNAs that distinguish COVID-19 non-survivors from survivors and from ARDS patients as a disease-matched control, and whether some circRNA signatures are stable over time. To the best of our knowledge, this work is the first longitudinal circRNA study to compare these clinical groups and the first to use a precision-weighted combination of time-resolved expression estimates.

Previous longitudinal studies have investigated differential mRNA expression across different COVID-19 disease statuses (COVID-19 survival and non-survival) and compared them with that in ARDS as a disease-matched control. For example, in [14], the authors elucidated the dysregulation of interferon signaling, iron homeostasis, and erythrocyte function, which correlates with survival in COVID-19.

The primary aim of this study is to distinguish the circRNA expression pattern associated with COVID-19 outcome, and to determine whether it can serve as a biomarker for diagnosis and prognosis. In this study, host circRNA differential expression was evaluated across COVID-19 non-survivors and survivors, as well as patients with acute respiratory distress syndrome (ARDS) as disease-matched controls, using total RNA-seq from GSE273149 [14], sampled at Day 3 (Early timepoint) and from Days 7 to 10 (Late window). The analysis revealed greater effect sizes and counts at the Late timepoint and a concise, time-stable circRNA set that discriminated COVID-19 non-survivors from ARDS controls and from survivors. To the best of our knowledge, this is the first longitudinal study of host circRNAs across these cohorts and the first precision-weighted analysis combining Early and Late timepoints.

## 2. Materials and Methods

### 2.1. Data Acquisition and Quality Control

Total RNA-seq data and sample annotations were retrieved from the publicly available GEO accession GSE273149. The RNA-seq data include three disease groups—ARDS controls, COVID-19 non-survivors, and COVID-19 survivors—and two analysis stages: Early (Day 3) and Late (Days 7–10 pooled). Peripheral blood mononuclear cells (PBMCs), total RNA isolation, and sequencing are described in the original publication [14]. The number of unique individuals and samples per disease group and timepoint, and the overlap between the Early and Late stages, are presented in Table S1b.

After downloading the FASTQ files, the quality of the raw RNA-seq reads was assessed using FastQC (v0.11.9). Adapter sequences and low-quality bases were removed with Trimmomatic (v0.39) before downstream analyses were conducted. Across all samples, quality metrics were strong, with a median Phred score of >30, balanced nucleotide composition, and no detectable adapter contamination. Table S1c shows the sequence depth and STAR alignment summary per sample.

### 2.2. CircRNA Detection and Annotation

Reads were mapped to the human reference genome (GRCh38) with STAR (v2.7.11b), which was configured for chimeric junction discovery. Putative circRNAs were then extracted and annotated with CIRCexplorer2 (v2.3.8), using Gencode v39-derived refFlat annotations to assign genomic context.

### 2.3. Quantification and Differential Analysis

CircRNA candidates with ≥2 back-splice junction reads were filtered to reduce single-read artifacts while preserving sensitivity for low-abundance circRNAs, particularly since the dataset was obtained from a total RNA library preparation, which is not enriched for circRNAs. Differential expression was assessed with DESeq2 in R (v4.4.2), while tidyverse was used for data wrangling, and figures were generated with ggplot2. Non-parametric tests were applied for individual comparisons, and group differences were evaluated via PERMANOVA.

### 2.4. Two-Timepoint, Precision-Weighted Analysis and Direction Flip Analysis

For each disease status, differential expression was estimated separately at the Early (Day 3) and Late (Days 7–10) timepoints using DESeq2 on the primary dataset, for which a filter of ≥10 raw counts in ≥20% of samples was used to reduce extreme sparsity and limit single-sample effects; in addition, size factors were computed, and QC was applied consistently across disease statuses. From each timepoint, the log_2_ fold change and its standard error were obtained. To emphasize effects that were stable across time, the two timepoint estimates were combined via fixed-effects, inverse-variance (precision-weighted) summarization, yielding a single effect estimate and a *p*-value for each circRNA. Multiple testing was controlled via Benjamini–Hochberg FDR, and the results were categorized as significant (FDR < 0.05) or suggestive (0.05–0.10), with |log_2_FC| ≥ 1 used as an interpretive guide. Directional concordance between timepoints was required for promotion in the combined analysis; sign-discordant features were flagged as time-dependent and described separately.

This approach was chosen to reflect the biological processes expected in PBMCs during severe COVID-19, where many disease-related shifts are state-like and persist across several days. By combining Early and Late estimates with precision weighting, information from both timepoints was leveraged while assigning greater influence to the more precise estimate, thereby increasing power and stabilizing effect sizes relative to separate analyses. At the same time, directional concordance was required, so signals that flipped signs over time were not promoted by the combined analysis and were instead treated as time-dependent effects. This strategy reduced over-interpretation of transient fluctuations; prioritized circRNAs with durable, biologically plausible differences; and provided a compact set of candidates for validation while preserving transparency through full single-timepoint results (shown in the Supplementary Materials). Additionally, given the nature of the dataset in this study, mixed-effects or time-by-group interaction models were not used, as sampling was incomplete across subjects, resulting in an unbalanced repeated-measures design; therefore, a precision-weighted analysis was conducted. Also, because some patients contributed samples from both Day 7 and Day 10 to the pooled Late timepoint, and DESeq2 does not explicitly model within-subject correlation, pooled Late results were treated as an inexact phase-level summary. Using a precision-weighted analysis was more appropriate, as it does not rely on a single pooled Late *p*-value; instead, it combines Early and Late effect estimates and requires directional concordance.

For flip analysis, circRNA log2 fold changes were estimated separately at Early and Late timepoints, and their signs were compared. CircRNAs were classified as direction-flipping if the Early and Late estimates had opposite signs (Early log2 fold change × Late log2 fold change < 0) and showed at least nominal evidence of differential expression at one or both timepoints (q < 0.1). These transcripts were then presented on plots of Early versus Late log2 fold change to visualize inversions in direction across time. Flipping was performed at the Early (Day 3) and Late (Day 7 + 10) timepoints; Day 7 and Day 10 were not evaluated separately because Day 10 sampling was incomplete across subjects and groups, which would affect the analysis and reduce its power. A low circRNA count can lead to a flip change, so only circRNAs with baseMean > 15 were employed in downstream network construction and interpretation; however, the full flip list is reported for transparency.

### 2.5. circRNA–miRNA–Immune Cell Interaction Network

Candidate circRNAs were first selected from the precision-weighted differential expression analysis; we selected those with a baseMean greater than 15 and significant, time-consistent differences between COVID-19 non-survivors and the comparison groups. For each chosen circRNA, predicted miRNA interactors were retrieved from circAtlas 3.0 [13] and then mapped to immune cell types using MiEAA [15], focusing on enrichment in T-cells, B-cells, NK-cells, and monocytes. The resulting circRNA–miRNA–cell relationships were assembled into a tripartite network, and Cytoscape 3.10.3 was used to generate the circRNA–miRNA–immune cell interaction figures.

## 3. Results

### 3.1. circRNA Detection and Quantification

Total RNA-seq data were retrieved from GEO (GSE273149). Samples from Day 3 were designated Early, and samples from Days 7 to 10 were pooled and designated Late, across three disease groups: ARDS controls, COVID-19 non-survivors, and COVID-19 survivors. For each sample, the number of expressed circRNAs was counted and summarized by group and stage (Table S1a). Group differences in circRNA counts were tested separately for each stage via pairwise Wilcoxon rank-sum tests, and *p*-values were adjusted using the Benjamini–Hochberg method. No significant between-group differences were observed at either stage, except for a Late-stage difference between ARDS controls and COVID-19 non-survivors (Table S1a).

**Figure 1:**
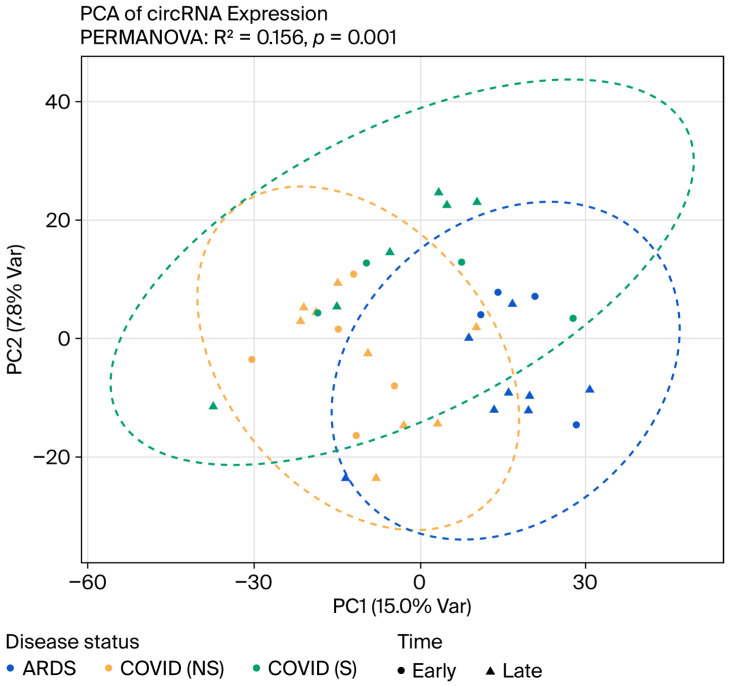
Principal component analysis (PCA) of circRNA expression profiles of different disease statuses. ARDS samples tend to occupy a region distinct from COVID-19 groups, while COVID-19 non-survivors (NS) and survivors (S) show only partial separation with visible overlap. PERMANOVA confirmed a significant effect of disease status (R^2^ = 0.156; *p* = 0.001).

The analysis of global circRNA expression structure separated samples primarily by disease status rather than by time. In the principal component analysis (PCA) (PC1 = 15.0% variance; PC2 = 7.8%), shown in Figure 1, ARDS samples were found to primarily occupy a region distinct from COVID-19 groups, while COVID-19 non-survivors and COVID-19 survivors show only partial separation with visible overlap. The Early and Late points show considerable overlap within each disease group, suggesting only a minimal effect of disease stage on the overall circRNA expression profile. PERMANOVA confirmed a significant effect of disease status (R^2^ = 0.156; *p* = 0.001) consistent with this visual pattern, as shown in Figure 1. These results indicate that disease status is the dominant driver of circRNA variation. In contrast, the Early-to-Late shift contributes comparatively little to between-sample differences at the whole-transcriptome level. Despite this, separating Early and Late data remains essential to evaluating circRNAs with distinct temporal behavior, including those that emerge, wane, or reverse in direction over time—patterns that would be blurred in a time-collapsed analysis.

### 3.2. circRNA Differential Expression: Single-Timepoint Results

Differential expression was assessed separately in the Early and Late stages for each clinical condition. Regarding Early differences between COVID-19 non-survivors and ARDS controls, 21 significant (FDR < 0.05) and 12 suggestive circRNAs (0.05 ≤ FDR < 0.10) were identified; see the volcano plot in Figure S1A and full results in Table S2. Regarding the Late stage, 19 significant and 30 suggestive circRNAs were detected, summarized in Table S3 and visualized in Figure S1B.

For COVID-19 survivors vs. ARDS controls, the Early-stage analyses yielded two significant circRNAs (Figure S2A and Table S4). In the Late stage, the signal increased substantially, with 75 essential and 15 suggestive circRNAs (Figure S2B, Table S5).

Regarding the comparison of COVID-19 non-survivors vs. survivors, Early-stage analyses indicated four significant circRNAs (Figure S3A and Table S6), while in the Late stage, 14 essential and 10 suggestive circRNAs were identified (Figure S3B and Table S7).

### 3.3. circRNA Differential Expression: Precision-Weighted Integration Across Timepoints

Because several effects were directionally consistent over time but marginal at individual timepoints, precision-weighted integration across timepoints was applied to identify time-consistent circRNA differences. This approach increased statistical power by pooling information from the Early and Late stages while weighting estimates by their precision, thereby prioritizing robust, reproducible signals [7,8].

**Figure 2:**
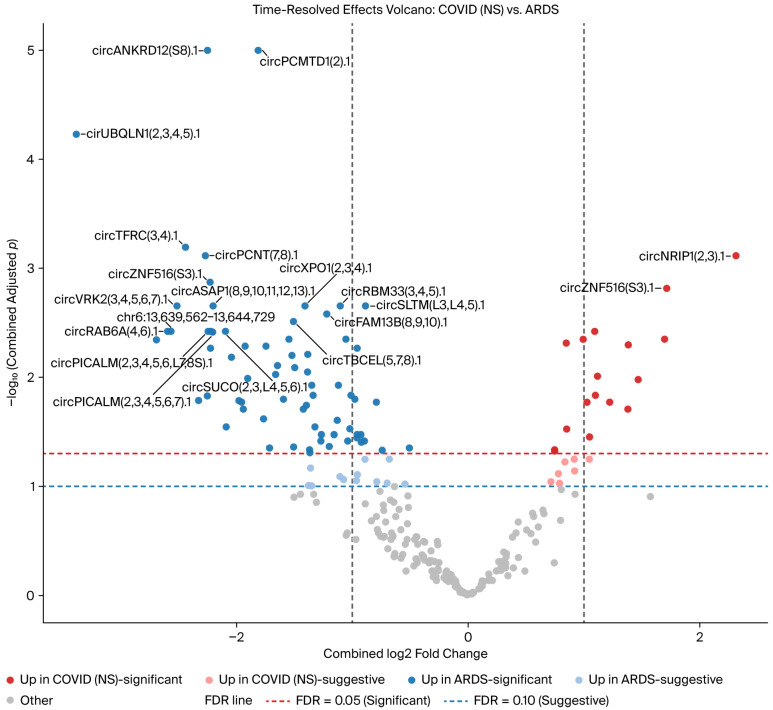
Volcano plot of differential circRNA expression between COVID-19 non-survivors and ARDS controls. Each point represents a circRNA, plotted according to the log_2_ fold change and the negative log_10_-adjusted *p*-value. Significant (adjusted *p* < 0.05) and suggestive circRNAs (adjusted *p* < 0.1) are indicated, with select circRNAs labeled. Upregulated circRNAs in each group are color-coded, and thresholds for significance and suggestiveness are shown.

**Figure 3:**
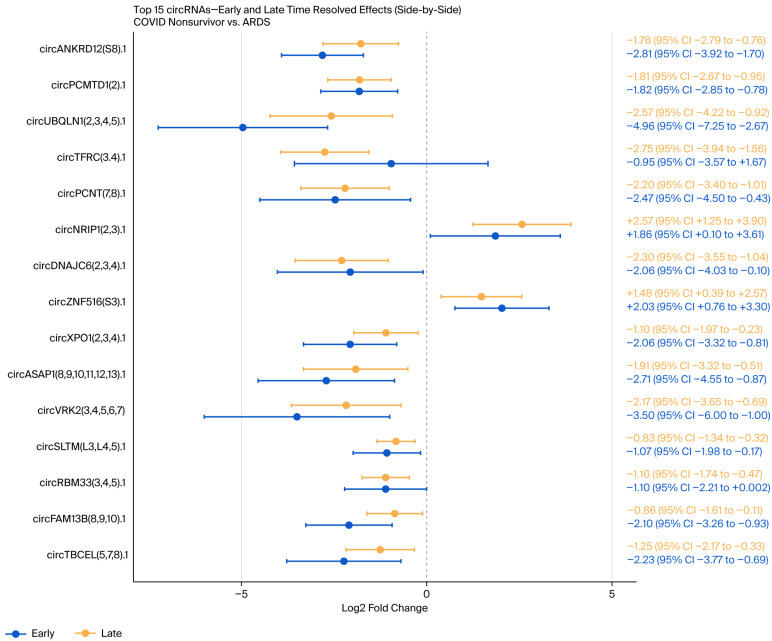
Forest plot of the 15 circRNAs with the highest 95% confidence intervals with Early and Late time-resolved effects for COVID-19 non-survivors and ARDS controls.

A total of 24 circRNAs were increased in COVID-19 non-survivors (17 statistically significant and 7 suggestive), while 86 were increased in the ARDS controls (69 statistically significant and 12 suggestive), with an FDR < 0.05 considered statistically significant, while a value of 0.05–0.10 was considered suggestive (Table S8). A time-resolved, precision-weighted volcano plot summarizing combined Early and Late effects is shown in Figure 2, and the 15 circRNAs with the highest 95% confidence intervals are displayed in the forest plot in Figure 3.

**Figure 4:**
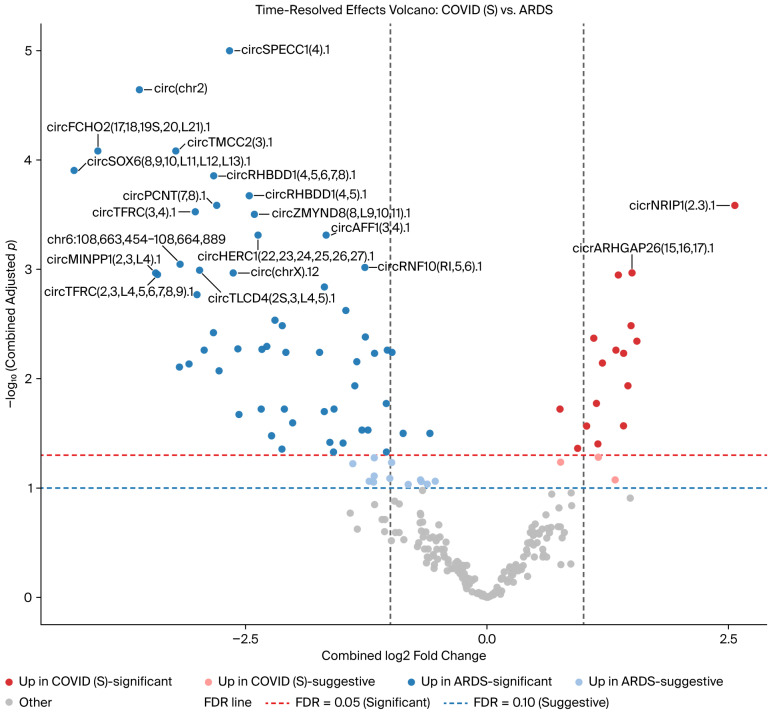
Volcano plot of differential circRNA expression between COVID-19 survivors and ARDS controls. Each point represents a circRNA, plotted according to the log_2_ fold change and the negative log_10_-adjusted *p*-value. Significant (adjusted *p* < 0.05) and suggestive circRNAs (adjusted *p* < 0.1) are indicated, with select circRNAs labeled. Upregulated circRNAs in each group are color-coded, and thresholds for significance and suggestiveness are shown.

**Figure 5:**
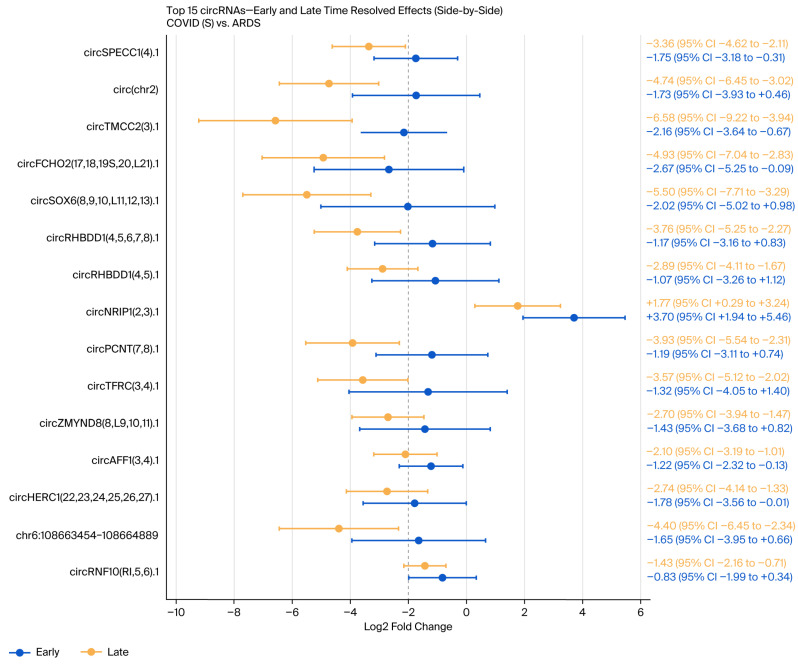
Forest plot of the 15 circRNAs with the highest 95% confidence intervals with Early and Late time-resolved effects for COVID-19 survivors and ARDS controls.

Regarding the comparison of COVID-19 survivors vs. ARDS controls, 16 circRNAs were increased in COVID-19 survivors at FDR < 0.05, and 3 were suggestive (FDR 0.05–0.10), whereas 56 circRNAs were increased in ARDS at FDR < 0.05, with an additional 12 being suggestive (Table S9). The time-resolved, precision-weighted volcano plot summarizing combined Early and Late effects is shown in Figure 4, and the 15 circRNAs with the highest 95% confidence intervals are displayed in the forest plot in Figure 5.

**Figure 6:**
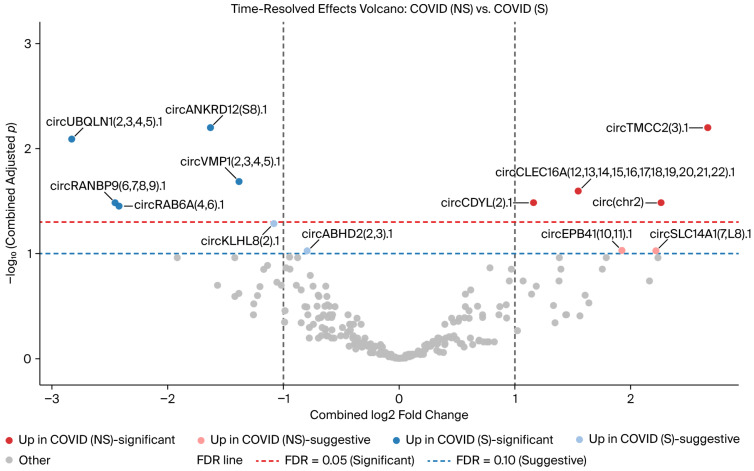
Volcano plot of differential circRNA expression between COVID-19 non-survivors and survivors. Each point represents a circRNA, plotted according to the log_2_ fold change and the negative log_10_-adjusted *p*-value. Significant (adjusted *p* < 0.05) and suggestive circRNAs (adjusted *p* < 0.1) are indicated, with select circRNAs labeled. Upregulated circRNAs in each group are color-coded, and thresholds for significance and suggestiveness are shown.

**Figure 7:**
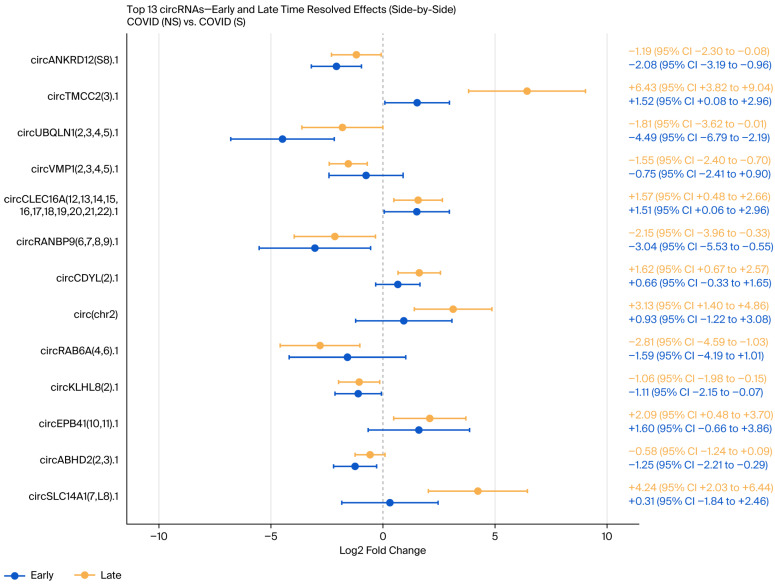
Forest plot of the 13 circRNAs with the highest 95% confidence intervals with Early and Late time-resolved effects for COVID-19 non-survivors and survivors.

Regarding the comparison of COVID-19 non-survivors vs. survivors, four circRNAs were increased in COVID-19 non-survivors at FDR < 0.05, while an additional two were suggestive (0.05–0.10). In contrast, five circRNAs were increased in COVID-19 survivors at FDR < 0.05, while two were suggestive (Table S10). A time-resolved, precision-weighted volcano plot summarizing combined Early and Late effects is shown in Figure 6, and the 13 circRNAs with highest 95% confidence intervals are displayed in the forest plot in Figure 7.

### 3.4. COVID-19 (NS) vs. COVID-19 (S): Top circRNA–miRNA–Immune Cell Network

Using precision-weighted integration across timepoints for circRNA differential expression, two circRNAs, circTMCC2(3).1 and circCDYL(2).1, were identified as significantly upregulated in the COVID-19 non-survivor (NS) cohort, each with baseMean > 15; conversely, circANKRD12(S8).1 was identified as significantly upregulated in the COVID-19 survivor (S) cohort. For candidate reporting, a baseMean > 15 threshold was applied to ensure adequate average normalized abundance, reduce low-count stochastic variability, stabilize effect-size estimates under multiple testing, and enhance biological interpretability. The COVID-19 (NS) vs. COVID-19 (S) comparison was selected for clinical relevance, as mortality-linked differences were isolated, enabling the derivation of candidate prognostic markers and potentially actionable pathways.

**Figure 8:**
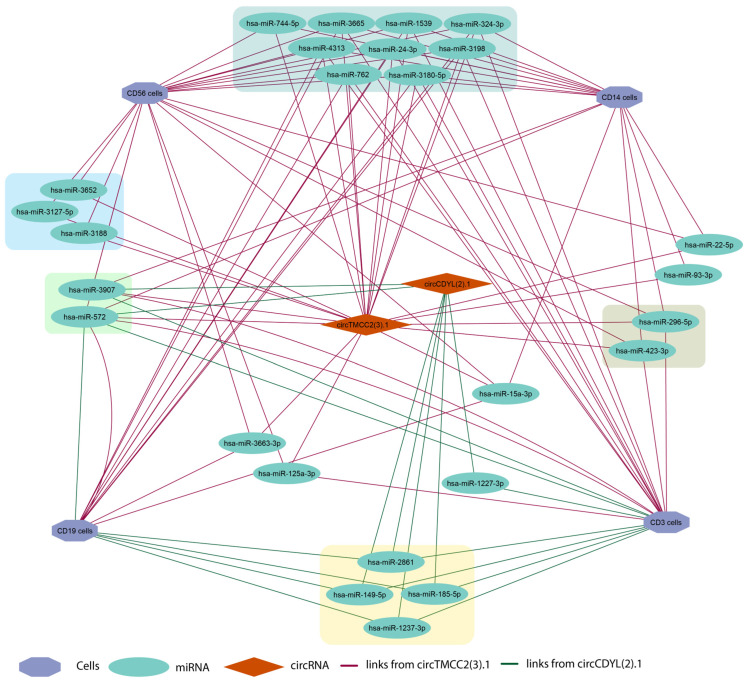
circTMCC2(3).1–circCDYL(2).1–miRNA–cell network showing predicted interactions between two circRNAs and immune cell-specific miRNAs. Hexagons represent immune cell subsets (CD3, CD14, CD19, and CD56), green ovals represent miRNAs, and orange diamonds represent circRNAs (circTMCC2(3):1 and circCDYL(2):1). Magenta edges indicate links between circTMCC2(3):1 and its predicted miRNA targets, and dark-green edges indicate links between circCDYL(2):1 and its predicted targets. Colored rectangles highlight clusters of miRNAs associated with the same circRNA–cell combinations.

circAtlas 3.0 was used to predict which miRNAs might bind to circRNA [13]. The MiEAA database was used to detect both potential miRNA and immune cells, since the circRNA data was obtained from PBMCs only; data on CD56 (NK-cells), CD3 (T-cells), CD19 (B-cells), and CD14 (mononucleates) were used [15]. Figure 8 shows the circTMCC2(3).1–circCDYL(2).1–miRNA–cell network. For circANKRD12(S8).1, the miRNAs retrieved from circAtlas 3.0 showed no enrichment for immune cell signatures in the miEAA database.

### 3.5. Flipping of circRNA Direction over Time

**Figure 9:**
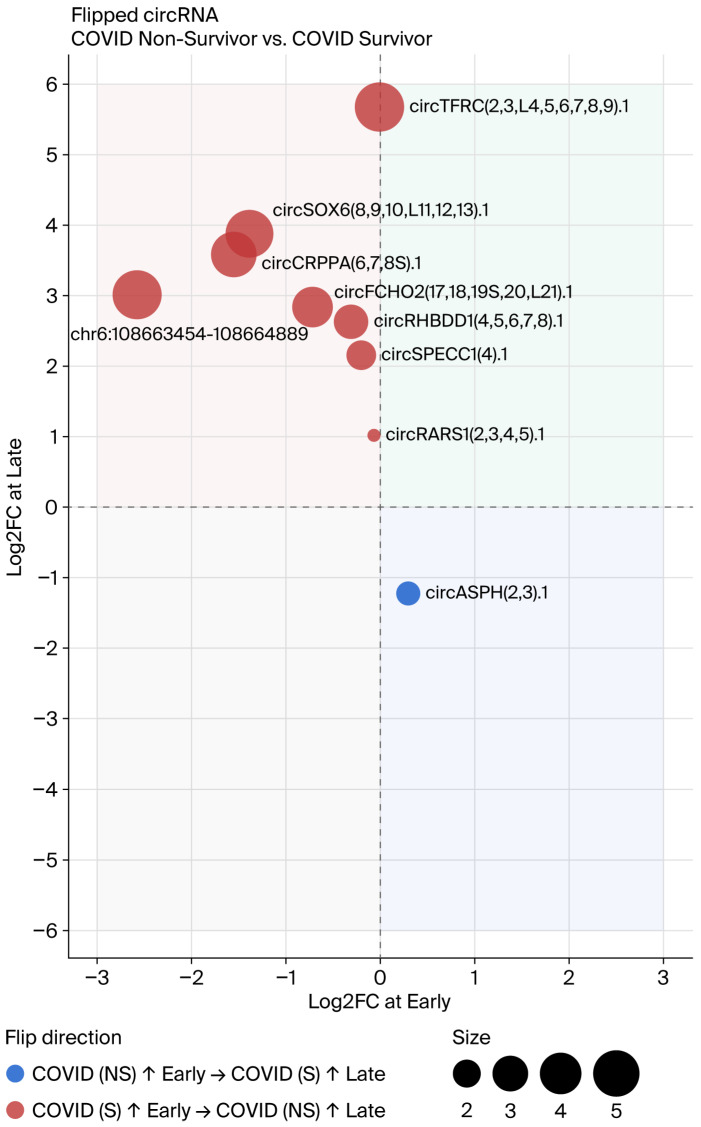
Flipped circRNAs in COVID-19 non-survivors vs. survivors.

**Figure 10:**
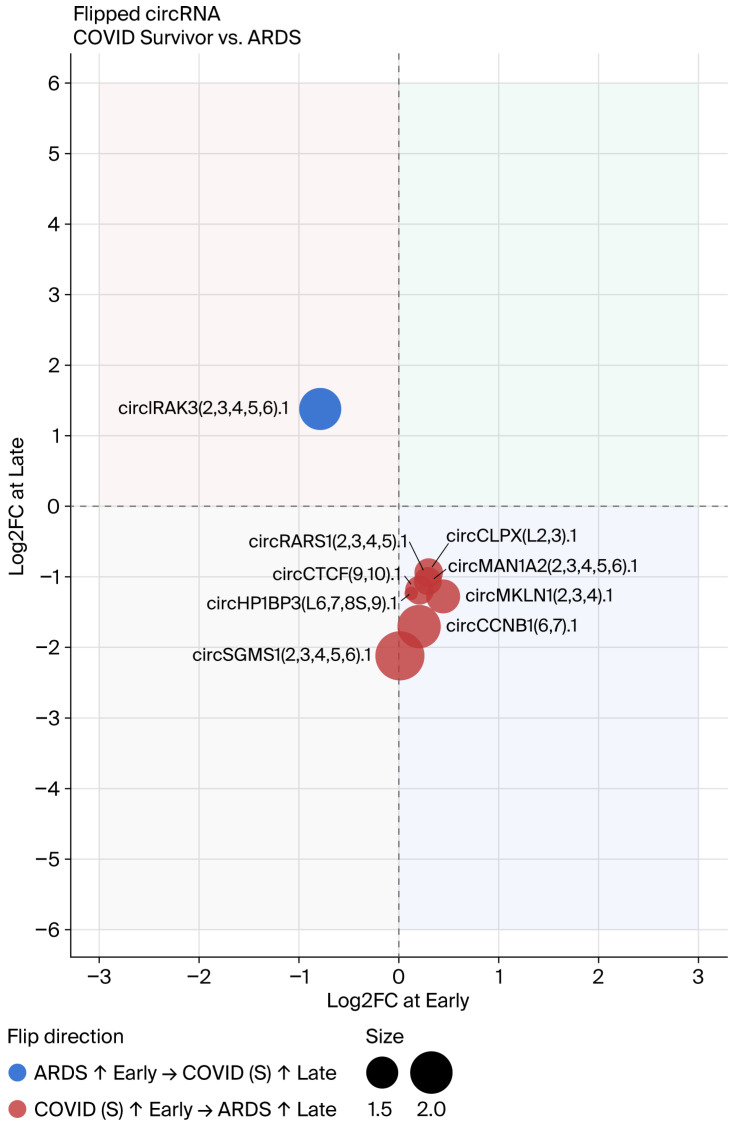
Flipped circRNAs in COVID-19 survivors vs. ARDS controls.

**Figure 11:**
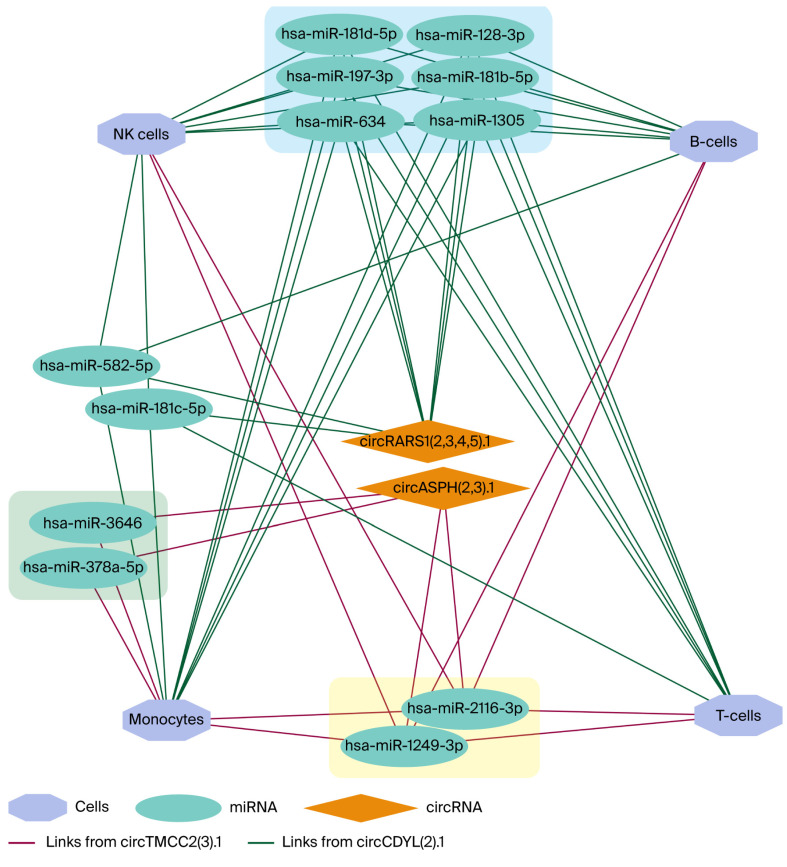
Flipped circRNA circASPH(2,3).1–circRARS1(2,3,4,5).1–miRNA–cell network depicting predicted interactions between circASPH(2,3):1, circRARS1(2,3,4,5):1, and immune cell-specific miRNAs. Hexagons represent immune cell subsets (NK-cells, B-cells, T-cells, and monocytes), teal ovals represent miRNAs, and orange diamonds represent circRNAs. Dark-green edges indicate predicted links from circRARS1(2,3,4,5):1, whereas magenta edges indicate links with circASPH(2,3):1. Colored rectangles highlight clusters of miRNAs that are jointly connected to the same circRNA–cell combinations.

A subset of circRNAs exhibited a time-dependent reversal, which was higher in one group at the Early timepoint but higher in the other at the Late timepoint. Flipped circRNAs were detected in two comparisons: COVID-19 (NS) vs. COVID-19 (S), where nine flipped circRNAs were identified (four significant, five suggestive; Figure 9, Table 2), and COVID-19 survivors vs. ARDS controls, where nine flipped circRNAs were identified (four significant, five suggestive; Figure 10, Table 3). circAtlas 3.0 was used to determine which miRNA might bind to circRNA [13]. The MiEAA database was used to detect both potential miRNA and immune cells, since the circRNA data was obtained from PBMCs only; data on NK-cells, T-cells, B-cells, and mononucleates were used [15]. Figure 11 shows the flipped circRNA circASPH(2,3).1–circRARS1(2,3,4,5).1–miRNA–cell network. Only the two circRNA–miRNA networks show links to immune cells in the MiEAA database.

## 4. Discussion

This study presents a longitudinal examination of circRNA expression of PBMCs among COVID-19 non-survivors and survivors, as well as ARDS controls, to identify stable and dynamically fluctuating RNA signatures that relate to clinical outcomes. Though the principal component analysis showed that disease status, rather than sampling time, was the leading cause of global circRNA variation, the two-timepoint design was needed to analyze individual circRNA trajectories.

A precision-weighted analysis of Early and late timepoints improved the statistical power and focused on circRNAs that showed consistent directional changes, which resulted in a parsimonious list of strong candidates that distinguished non-survivors from both survivors and ARDS controls. Among them, circTMCC2(3).1 and circCDYL(2).1 were found to be reproducibly increased in non-survivors, and circANKRD12(S8).1 was increased in survivors. Even though these circRNAs have not been mechanistically associated with the pathology of COVID-19, their temporal reproducibility suggests that they can indicate long-lasting changes in immune or inflammatory conditions associated with mortality risk.

To put these findings into a biological context, predicted circRNA–miRNA interactions were projected onto immune-cell-specific enrichment profiles, and circTMCC2(3).1 and circCDYL(2).1 were identified to be connected with miRNAs of major PBMC subsets—T cells, B cells, monocytes, and NK cells—indicating that they may be involved in systemic immune dysregulation in severe disease. On the other hand, circANKRD12(S8).1 was not specifically enriched in immune cells, suggesting that it may have a different functional role.

Unlike these candidates, which are time-invariant, various circRNAs exhibited direction-flipping behavior, which is defined as opposite Early and Late log2 fold changes. circRARS1(2,3,4,5).1 and circASPH(2,3).1 were circRNAs that had immune-correlated miRNA changes, and it is possible that reversible changes happening over time are linked to changes in specific subsets of PBMCs rather than random noise.

Although this study offers several interesting observations, it has some limitations. Firstly, it was dependent on one cohort, which makes generalizability of the results difficult, and it could be insensitive to low-abundance transcripts because it employed total RNA-seq but not circRNA-enriched sequencing data. The reported suggestive (FDR 0.05–0.10) circRNAs should be analyzed with caution and considered hypothesis-driven circRNAs that require independent validation. Moreover, the circRNA–miRNA–immune cell networks are also merely computational predictions, even though the hypotheses are biologically plausible. To determine mechanistic relevance, it will be necessary to conduct experimental validation, especially in sorted immune cell populations.

These results indicate that longitudinal information should be incorporated with precision-weighted techniques to identify biologically meaningful circRNA signatures in severe cases of COVID-19. The resulting direction-flipping and time-consistent circRNAs should be employed as targets in future translational medical research, which could lead to the development of biomarkers or elucidation of host response mechanisms in viral pneumonia.

Future studies should focus on validating the top candidates using targeted measurements, such as RT-qPCR, in longitudinal cohorts, and test whether these circRNAs can be used to clinically predict COVID-19 severity. Additionally, investigating the circRNA pattern across different SARS-CoV-2 variants will provide further mechanistic insight by distinguishing conserved responses from variant-specific effects. Moreover, the association between circRNA patterns and immune cytokine patterns may play an important role in COVID-19 pathogenesis, making circRNA patterns a valuable factor in multivariable prediction models for COVID-19 severity.

## 5. Conclusions

This study uses a two-timepoint, precision-weighted analysis of PBMC circRNA differential expression to distinguish the circRNA pattern associated with outcomes in COVID-19 non-survivors and survivors, as well as ARDS controls. It highlights a small set of time-consistent circRNAs associated with mortality, along with direction-flipping candidates that exhibit dynamic behavior over time. Integration with miRNA enrichment profiles in immune cell subsets placed these circRNAs within plausible immune regulatory contexts, although the networks remain inferential and require experimental validation. These findings suggest that blood circRNAs may provide clinically relevant biomarkers of the host response to severe COVID-19 and ARDS, and illustrate a general framework for time-resolved circRNA analysis in other diseases.

## Figures and Tables

**Table 1:** Association of circRNA with COVID-19 according to different studies.

circRNA Name	circAtlas and Uniform ID *	Type of Study (Longitudinal vs. Non-Longitudinal)	Potential Function/Associated Pathway	Expression in COVID-19	Sample Type	Reference
hsa_circ_0127052 (circRasGEF1B)	NA; chr4:82377804-82380668	Non-longitudinal	Innate immune regulation (suspected miRNA sponge); proposed diagnostic biomarker	Upregulated	Nasopharyngeal swab. Sample collected during the Delta variant outbreak	[9]
hsa_circ_100783 (circHIPK3)	NA; chr11:33307958–33350179	Non-longitudinal	Immune modulation and proliferation: proposed diagnostic biomarker	Upregulated	Nasopharyngeal swab. Sample collected during the Delta variant outbreak	[6,9]
hsa_circ_0004812	hsa-NINL_0001 (circNINL(9,10).1)	Non-longitudinal	Cytokine storm mediator that functions via miR-1287-5p/IL6R/RIG-I axis	Upregulated	PBMC	[10]
hsa_circ_0000479	hsa- EPSTI1_0003 (circEPSTI1(2,3,4,5,6S).1)	Non-longitudinal	Immune response regulator that functions via miR-149-5p/RIG-I/IL-6 axis	Upregulated	PBMC	[3,11]
hsa_circ_0080942	hsa-PCLO_0007 (circPCLO(2,3).1)	Non-longitudinal	Cytokine-storm network regulator (ceRNA sponging cytokine-related miRNAs)	Upregulated (predicted)	Whole blood samples and lung tissue samples	[12]
hsa_circ_0080135 (circTNS3)	NA; chr7:47341783-47409218	Non-longitudinal	Cytokine-storm network regulator (Competing Endogenous RNA sponging cytokine-related miRNAs)	Upregulated (predicted)	Whole blood samples and lung tissue samples	[12]

* circAtlas and uniform ID were obtained from circAtlas 3.0 [13]. NA = not available; the circRNAs found in some studies are not listed in circAtlas 3.0; for these, the chromosome location is given instead—PBMC = Peripheral blood mononuclear cells.

**Table 2:** Flipped circRNAs: COVID-19 (NS) vs. COVID-19 (S).

circAtlas ID	Uniform ID	Upregulated, Early	Upregulated, Late	log2FC, Early	log2FC, Late	BaseMean, Early	BaseMean, Late	BaseMean, mean	FDR, Day 3	FDR, Late	
hsa-TFRC_0004	circTFRC(2,3,L4,5,6,7,8,9).1	COVID-19 (S)	COVID-19 (NS)	−0.11	5.57	12.50	17.89	15.20	0.976	0.004	Significant
hsa-SOX6_0034	circSOX6(8,9,10,L11,12,13).1	COVID-19 (S)	COVID-19 (NS)	−1.49	3.90	3.61	4.14	3.88	0.801	0.025	Significant
hsa-SPECC1_0001	circSPECC1(4).1	COVID-19 (S)	COVID-19 (NS)	−0.15	2.04	89.11	99.40	94.25	0.962	0.033	Significant
hsa-RHBDD1_0003	circRHBDD1(4,5,6,7,8).1	COVID-19 (S)	COVID-19 (NS)	−0.39	2.41	10.37	10.59	10.48	0.892	0.040	Significant
hsa-RARS_0012	circRARS1(2,3,4,5).1	COVID-19 (S)	COVID-19 (NS)	−0.10	1.14	10.83	11.13	10.98	0.970	0.042	Significant
chr6:108663454-108664889	chr6:108663454-108664889	COVID-19 (S)	COVID-19 (NS)	−2.58	3.03	4.50	5.20	4.85	0.593	0.067	Suggestive
hsa-AC090094_0001	circASPH(2,3).1	COVID-19 (NS)	COVID-19 (S)	0.23	−1.45	22.27	18.72	20.49	0.917	0.067	Suggestive
hsa-ISPD_0004	circCRPPA(6,7,8S).1	COVID-19 (S)	COVID-19 (NS)	−1.46	3.35	3.64	3.09	3.37	0.738	0.079	Suggestive
hsa-FCHO2_0068	circFCHO2(17,18,19S,20,L21).1	COVID-19 (S)	COVID-19 (NS)	−0.75	3.03	4.49	5.12	4.80	0.832	0.079	Suggestive

**Table 3:** Flipped circRNAs: COVID-19 (S) vs. ARDS.

circAtlas ID	Uniform ID	Upregulated, Early	Upregulated, Late	log2FC, Early	log2FC, Late	BaseMean, Early	BaseMean, Late	BaseMean, mean	FDR, Day 3	FDR, Late	
hsa-MAN1A2_0008	circMAN1A2(2,3,4,5,6).1	COVID-19 (S)	ARDS	0.19	−1.17	16.20	13.96	15.08	0.96	0.01	Significant
hsa-RARS_0012	circRARS1(2,3,4,5).1	COVID-19 (S)	ARDS	0.14	−1.00	10.83	11.13	10.98	0.97	0.03	Significant
hsa-CCNB1_0001	circCCNB1(6,7).1	COVID-19 (S)	ARDS	0.26	−1.82	4.03	3.50	3.76	0.97	0.05	Significant
hsa-CLPX_0007	circCLPX(L2,3).1	COVID-19 (S)	ARDS	0.22	−1.16	11.56	12.96	12.26	0.96	0.05	Significant
hsa-MKLN1_0004	circMKLN1(2,3,4).1	COVID-19 (S)	ARDS	0.41	−1.16	7.39	6.31	6.85	0.91	0.06	Suggestive
hsa-CTCF_0008	circCTCF(9,10).1	COVID-19 (S)	ARDS	0.20	−1.17	5.68	7.39	6.53	0.97	0.08	Suggestive
hsa-IRAK3_0003	circIRAK3(2,3,4,5,6).1	ARDS	COVID-19 (S)	−0.84	1.16	6.40	5.93	6.17	0.73	0.09	Suggestive
hsa-SGMS1_0007	circSGMS1(2,3,4,5,6).1	COVID-19 (S)	ARDS	0.10	−2.36	2.68	2.94	2.81	0.99	0.096	Suggestive
hsa-HP1BP3_0004	circHP1BP3(L6,7,8S,9).1	COVID-19 (S)	ARDS	0.09	−1.04	8.11	8.16	8.13	0.98	0.099	Suggestive

## Data Availability

All data analyzed in this study are publicly available from the Gene Expression Omnibus (GEO) under the accession number GSE273149. The datasets can be accessed at https://www.ncbi.nlm.nih.gov/geo/ (last accessed on 8 November 2025) using the provided accession numbers.

## Supplementary Materials

The following supporting information can be downloaded at https://www.mdpi.com/article/10.3390/genes17010034/s1, Figure S1: Volcano plot circRNA differential expression COVID (NS) vs. ARDS early and late. Figure S2: Volcano plot circRNA differential expression COVID (S) vs. ARDS early and late. Figure S3: Volcano plot circRNA differential expression COVID (NS) vs. COVID (S) early and late. Table S1a and b: Summary Statistics for circRNAs detected per group per day. Table S1c: Sequencing depth and STAR alignment summary per sample title. Table S2: Top differentially expressed circRNAs between COVID non-survival and ARDS at early (Day 3) stage. Table S3: Top differentially expressed circRNAs between COVID non-survival and ARDS at late (Day 7 + 10) stage. Table S4: Top differentially expressed circRNAs between COVID survival and ARDS at early (Day 3) stage. Table S5: Top differentially expressed circRNAs between COVID survival and ARDS at late (Day 7 + 10) stage. Table S6: Top differentially expressed circRNAs between COVID non-survival and COVID survival at early (Day 3) stage. Table S7: Top differentially expressed circRNAs between COVID non-survival and COVID survival at late (Day 7 + 10) stage. Table S8: Time-Resolved circRNA Differences (Early + Late)—COVID Non-Survivor vs. ARDS. Table S9: Time-Resolved circRNA Differences (Early+ Late)—COVID Survivor vs. ARDS. Table S10: Time-Resolved circRNA Differences (Early + Late)—COVID Non-Survivor vs. COVID Survivor.

## Abbreviations

The following abbreviations are used in this manuscript:

ARDS	Acute respiratory distress syndrome
COVID-19	Coronavirus disease 2019
SARS-CoV-2	Severe acute respiratory syndrome coronavirus 2
PBMC	Peripheral blood mononuclear cell
PBMCs	Peripheral blood mononuclear cells
circRNA	Circular RNA
miRNA	MicroRNA
mRNA	Messenger RNA
RNA-seq	RNA sequencing
GEO	Gene Expression Omnibus
FDR	False discovery rate
log2FC	Log2 fold change
PCA	Principal component analysis
PC	Principal component
PERMANOVA	Permutational multivariate analysis of variance
NAAT	Nucleic acid amplification test
CRP	C-reactive protein
IL-6	Interleukin 6
IFN-γ	Interferon gamma
IgG	Immunoglobulin G
IgA	Immunoglobulin A
NK-cell	Natural killer cell
RIG-I	Retinoic acid-inducible gene I
ADAR	Adenosine deaminases acting on RNA.
APOBEC	Apolipoprotein B mRNA-editing enzyme catalytic polypeptide-like
DESeq2	Differential expression analysis tool in R (DESeq2 package)
CD3	T-cell marker (CD3)
CD14	Monocyte marker (CD14)
CD19	B-cell marker (CD19)
CD56	NK-cell marker (CD56)
bp	Base pair
nt	Nucleotide
CI	Confidence interval
BH	Benjamini–Hochberg
R^2^	Coefficient of determination
