# Supplementary material for: circRNA Signatures Distinguishing COVID-19 Outcomes and Acute Respiratory Distress Syndrome: A Longitudinal, Two-Timepoint, Precision-Weighted Analysis of a Public RNA-Seq Cohort
Source: Genes (Basel). 2025 Dec 30;17(1):34. doi: 10.3390/genes17010034 (PMC12841326; doi:10.3390/genes17010034)
Supplement: Supplementary file 1 [file genes-17-00034-s001.zip › Table S4 Top differentially expressed circRNAs between COVID survival and ARDS at early (Day 3) stage.pdf]

Table S4: Top differentially expressed circRNAs between COVID survival and ARDS at early (Day 3) stage

circAtlas ID	Uniform ID	Gene name	baseMean	Log2Fold Change	lfcSE	Stat	pvalue	padj
chr21:15014343-15043574	hsa-NRIP1_0002	circNRIP1(2,3).1	NRIP1	12.98	3.70	0.90	4.13	0.00004
chr2:106158057-106166083	hsa-UXS1_0001	circUXS1(2,3S,4,5).1	UXS1	5.88	3.37	0.83	4.08	0.00004

baseMean: Average expression level across all samples. log2FoldChange: Log2-transformed fold change between two conditions, Negative value means downregulated in COVID non-survival and positive means upregulated in COVID survival. lfcSE: log2 fold change of standard error. Stat: Statistical test value for differential expression. pvalue: Raw p-value from the statistical test. padj: Adjusted p-value (corrected for multiple testing).
