# Supplementary material for: circRNA Signatures Distinguishing COVID-19 Outcomes and Acute Respiratory Distress Syndrome: A Longitudinal, Two-Timepoint, Precision-Weighted Analysis of a Public RNA-Seq Cohort
Source: Genes (Basel). 2025 Dec 30;17(1):34. doi: 10.3390/genes17010034 (PMC12841326; doi:10.3390/genes17010034)
Supplement: Supplementary file 1 [file genes-17-00034-s001.zip › Table S5 Top differentially expressed circRNAs between COVID survival and ARDS at late (Day 7+10) stage.pdf]

Table S5: Top differentially expressed circRNAs between COVID survival and ARDS at late (Day 7+10) stage

circAtlas ID	Uniform ID	Gene name	baseMean	Log2Fold Change	lfcSE	Stat	pvalue	padj
hsa-ANKRD36BP2	circ(chr2)	ANKRD36BP2	10.80	-4.74	0.87	-5.42	0.0000001	0.00002
hsa-SPECC1_0001	circSPECC1(4).1	SPECC1	99.40	-3.36	0.64	-5.25	0.0000001	0.00002
hsa-SOX6_0034	circSOX6(8,9,10,L11,12,13).1	SOX6	4.14	-5.50	1.13	-4.88	0.0000010	0.00006
hsa-FECH_0013	circFECH(L2,3,4).1	FECH	2.50	-4.78	0.98	-4.86	0.0000012	0.00006
hsa-TMCC2_0001	circTMCC2(3).1	TMCC2	25.48	-6.58	1.35	-4.88	0.0000010	0.00006
hsa-RHBDD1_0003	circRHBDD1(4,5,6,7,8).1	RHBDD1	10.59	-3.76	0.76	-4.95	0.0000007	0.00006
hsa-PCNT_0003	circPCNT(7,8).1	PCNT	7.58	-3.93	0.82	-4.77	0.0000018	0.00007
hsa-TFRC_0004	circTFRC(2,3,L4,5,6,7,8,9).1	TFRC	17.89	-6.38	1.34	-4.76	0.0000019	0.00007
hsa-RHBDD1_0004	circRHBDD1(4,5).1	RHBDD1	12.11	-2.89	0.62	-4.66	0.0000032	0.00010
hsa-NUP54_0005	circNUP54(2,3).1	NUP54	3.03	-4.94	1.07	-4.61	0.0000039	0.00011
hsa-FCHO2_0068	circFCHO2(17,18,19S,20,L21).1	FCHO2	5.12	-4.93	1.07	-4.59	0.0000044	0.00012
hsa-MINPP1_0001	circMINPP1(2,3,L4).1	MINPP1	2.40	-5.05	1.12	-4.49	0.0000071	0.00016
hsa-TFRC_0013	circTFRC(3,4).1	TFRC	4.18	-3.57	0.79	-4.50	0.0000066	0.00016
hsa-PHLPP2_0024	circPHLPP2(L6,7,8).1	PHLPP2	2.04	-4.53	1.02	-4.44	0.0000090	0.00019
hsa-DNAJC6_0001	circDNAJC6(2,3,4).1	DNAJC6	9.90	-3.33	0.77	-4.34	0.0000143	0.00026
hsa-ISPD_0004	circCRPPA(6,7,8S).1	CRPPA	3.09	-5.16	1.19	-4.34	0.0000140	0.00026
hsa-ZMYND8_0005	circZMYND8(8,L9,10,11).1	ZMYND8	5.37	-2.70	0.63	-4.28	0.0000185	0.00032
hsa-ARHGAP26_0002	circARHGAP26(15,16,17).1	ARHGAP26	7.48	1.85	0.44	4.20	0.0000261	0.00042
circFOXO3	circFOXO3	FOXO3	5.20	-4.40	1.05	-4.19	0.0000274	0.00042
hsa-RCL1_0008	circRCL1(2,3).1	RCL1	5.33	-4.05	0.98	-4.13	0.0000355	0.00052
hsa-SLC14A1_0001	circSLC14A1(7,L8).1	SLC14A1	7.94	-4.64	1.13	-4.09	0.0000431	0.00059
hsa-TMEM56-RWDD3_0004	circTLCD4(2S,3,L4,5).1	TLCD4	19.36	-3.42	0.84	-4.07	0.0000476	0.00063
hsa-WWC3_0004	circ(chrX).12		5.64	-4.58	1.13	-4.04	0.0000533	0.00067
hsa-RNF10_0004	circRNF10(RI,5,6).1	RNF10	15.77	-1.43	0.37	-3.88	0.0001054	0.00127
hsa-HERC1_0035	circHERC1(22,23,24,25,26,27).1	HERC1	9.51	-2.74	0.72	-3.82	0.0001328	0.00154
hsa-AFF1_0001	circAFF1(3,4).1	AFF1	23.46	-2.10	0.56	-3.78	0.0001555	0.00173
hsa-VPS50_0002	circVPS50(13,14,15,16).1	VPS50	1.90	-4.28	1.14	-3.77	0.0001632	0.00175
hsa-ARHGEF12_0041	circARHGEF12(32,33L,34,35,36).1	ARHGFE12	2.52	-4.89	1.33	-3.69	0.0002255	0.00234
hsa-AKAP7_0001	circAKAP7(2,L3,4,5).1	AKAP7	5.16	-2.93	0.81	-3.63	0.0002864	0.00268
hsa-CDYL_0005	circCDYL(2).1	CDYL	60.55	-1.80	0.49	-3.64	0.0002696	0.00268
hsa-EZH2_0001	circEZH2(2,3).1	EZH2	3.20	-3.37	0.93	-3.63	0.0002792	0.00268

hsa-ZNF516_0005	circZNF516(S3).1	ZNF516	7.78	2.13	0.59	3.59	0.0003292	0.00290
hsa-AC090094_0001	circASPH(2,3).1	ASPH	18.72	1.84	0.51	3.59	0.0003296	0.00290
hsa-RAB3IP_0001	circRAB3IP(2,3).1	RAB3IP	4.13	-3.31	0.93	-3.55	0.0003784	0.00305
hsa-VRK1_0001	circVRK1(2,3).1	VRK1	31.70	-1.94	0.55	-3.54	0.0003983	0.00305
hsa-GMIP_0001	circGMIP(5,RI,6,7).1	GMIP	11.07	1.43	0.40	3.55	0.0003896	0.00305
hsa-DDI2_0008	circDDI2(5,6).1	DDI2	3.41	-3.82	1.08	-3.55	0.0003860	0.00305
hsa-RBM33_0009	circRBM33(3,4,5).1	RBM33	22.08	-1.29	0.37	-3.54	0.0003991	0.00305
hsa-RAB11FIP1_0002	circRAB11FIP1(2).1	RAB11FIP1	9.07	1.41	0.40	3.51	0.0004432	0.00330
hsa-VMP1_0001	circVMP1(2,3,4,5).1	VMP1	8.17	1.44	0.41	3.49	0.0004769	0.00346
hsa-SLC36A1_0002	circSLC36A1(2,3,4,5,6,7,8,9,10).1	SLC36A1	4.43	-4.15	1.19	-3.48	0.0004950	0.00350
hsa-NRXN3_0008	circNRXN3(11,12,13,14,15).1	NRXN3	2.41	-4.85	1.43	-3.39	0.0007083	0.00482
hsa-HERC1_0013	circHERC1(22,23,24L,25).1	HERC1	2.83	-3.22	0.95	-3.38	0.0007317	0.00482
hsa-EPB41_0021	circEPB41(10,11).1	EPB41	3.99	-2.72	0.80	-3.38	0.0007167	0.00482
hsa-TBCEL_0004	circTBCEL(3,4,5S,6,7S,8).1	TBCEL	5.33	-1.87	0.56	-3.32	0.0009149	0.00590
hsa-TMEM56-RWDD3_0001	circTLCD4(2S,3,4,5,6).1	TLCD4	6.54	-2.29	0.69	-3.31	0.0009392	0.00592
hsa-NAP1L4_0006	circNAP1L4(2,3,4,5S,6,7S,8,9,11,12,13,14).1	NAP1L4	5.35	-3.82	1.16	-3.28	0.0010335	0.00638
hsa-SOX6_0009	circSOX6(5,6).1	SOX6	3.63	-3.78	1.16	-3.25	0.0011409	0.00680
hsa-PCMTD1_0002	circPCMTD1(2).1	PCMTD1	26.84	-1.56	0.48	-3.25	0.0011491	0.00680
hsa-NCAPG_0005	circNCAPG(4,5).1	NCAPG	4.30	-3.01	0.96	-3.15	0.0016223	0.00941
hsa-RNF10_0006	circRNF10(5,6,7).1	RNF10	3.15	-2.69	0.86	-3.13	0.0017655	0.01004
hsa-RAB3D_0003	circRAB3D(3,4).1	RAB3D	7.53	2.22	0.72	3.09	0.0020328	0.01134
hsa-ARHGEF12_0042	circARHGEF12(31,32,33L,34,35,36).1	ARHGAP12	3.10	-3.66	1.21	-3.04	0.0024019	0.01290
hsa-NPRL3_0003	circNPRL3(L8,9).1	NPRL3	4.78	-2.75	0.90	-3.04	0.0023694	0.01290
hsa-MAN1A2_0008	circMAN1A2(2,3,4,5,6).1	MAN1A2	13.96	-1.16	0.39	-3.01	0.0026225	0.01369
hsa-FCHO2_0038	circFCHO2(20,21).1	FCHO2	8.42	-1.58	0.53	-3.01	0.0026436	0.01369
hsa-TOP1_0001	circTOP1(9,10,11,12,13).1	TOP1	6.48	-2.33	0.79	-2.94	0.0032332	0.01645
hsa-ANKRD13C_0027	circANKRD13C(4,5S,6,7S,8S,9).1	ANKRD13C	6.72	-1.57	0.54	-2.93	0.0033700	0.01685
hsa-SLC45A4_0002	circSLC45A4(2).1	SLC45A4	13.45	0.88	0.30	2.89	0.0038170	0.01876
hsa-SEC62_0004	circSEC62(3,L4,5,6,7).1	SEC62	10.31	-1.56	0.54	-2.88	0.0039671	0.01917
hsa-BACH1_0001	circBACH1(2,3,4).1	BACH1	3.68	-3.01	1.05	-2.87	0.0040412	0.01921
hsa-PCMTD1_0001	circPCMTD1(2,3).1	PCMTD1	2.57	-2.91	1.04	-2.81	0.0049958	0.02337
hsa-EP300_0003	circEP300(7,8,9).1	EP300	4.45	-2.22	0.80	-2.76	0.0057054	0.02626
hsa-RARS_0012	circRARS1(2,3,4,5).1	RARS1	11.13	-1.00	0.36	-2.75	0.0059663	0.02703
hsa-SLC37A3_0005	circSLC37A3(6,7).1	SLC37A3	9.05	1.43	0.53	2.70	0.0069872	0.03117

hsa-AURKA_0004	circAURKA(4,5,6).1	AURKA	3.49	-2.00	0.76	-2.63	0.0084452	0.03711
hsa-MYO9B_0005	circMYO9B(2).1	MYO9B	9.95	1.23	0.47	2.62	0.0087472	0.03786
hsa-CREBBP_0001	circCREBBP(2).1	CREBBP	8.08	-0.89	0.34	-2.60	0.0094475	0.04029
hsa-SWT1_0003	circSWT1(14,15,16).1	SWT1	3.95	-2.83	1.09	-2.58	0.0097954	0.04058
hsa-TMEM50A_0002	circTMEM50A(2,3,4,5,6).1	TMEM50A	2.72	-3.14	1.21	-2.59	0.0096598	0.04058
hsa-CLEC16A_0001	circCLEC16A(12,13,14,15,16,17,18,19,20,21,22).1	CLEC16A	8.08	-1.40	0.55	-2.53	0.0115256	0.04642
hsa-ILKAP_0001	circILKAP(6,7,8,9).1	ILKAP	5.79	-1.47	0.58	-2.53	0.0114274	0.04642
hsa-FBXW7_0005	circFBXW7(3,4).1	FBXW7	39.44	-0.75	0.30	-2.52	0.0118372	0.04702
hsa-CCNB1_0001	circCCNB1(6,7).1	CCNB1	3.50	-1.82	0.72	-2.51	0.0120005	0.04703
hsa-CLPX_0007	circCLPX(L2,3).1	CLPX	12.96	-1.16	0.46	-2.50	0.0125285	0.04844
hsa-CHSY1_0001	circCHSY1(2).1	CHSY1	6.88	1.11	0.45	2.46	0.0140357	0.05355
hsa-DOPEY2_0008	circDOP1B(20,21).1	DOP1B	5.87	1.80	0.73	2.45	0.0142189	0.05355
hsa-MVP_0002	circMVP(4,5).1	MVP	9.79	1.04	0.43	2.42	0.0157212	0.05845
hsa-PICALM_0001	circPICALM(2,3,4,5,6,7,8,9,10,11,12S).1	PICALM	4.71	-1.61	0.67	-2.40	0.0164617	0.06043
hsa-EP300_0036	circEP300(3,4,5,6,7,8,9).1	EP300	2.84	-2.76	1.16	-2.38	0.0172872	0.06267
hsa-MKLN1_0012	circMKLN1(2,3,4,5,6).1	MKLN1	6.31	-1.16	0.49	-2.37	0.0179228	0.06417
hsa-NRIP1_0002	circNRIP1(2,3).1	NRIP1	5.77	1.77	0.75	2.34	0.0190337	0.06731
hsa-ASH2L_0010	circASH2L(6,7,L8,9).1	ASH2L	2.94	-1.57	0.68	-2.32	0.0203774	0.07120
hsa-MAPK9_0009	circMAPK9(9,10,11).1	MAPK9	6.07	-1.19	0.52	-2.30	0.0211696	0.07309
hsa-CTCF_0008	circCTCF(9,10).1	CTCF	7.39	-1.17	0.52	-2.27	0.0232425	0.07930
hsa-IRAK3_0003	circIRAK3(2,3,4,5,6).1	IRAK3	5.93	1.16	0.53	2.20	0.0275382	0.09286
hsa-SGMS1_0007	circSGMS1(2,3,4,5,6).1	SGMS1	2.94	-2.36	1.08	-2.19	0.0288346	0.09612
hsa-CCSER2_0002	circCCSER2(4,5).1	CCSER2	8.04	1.07	0.49	2.17	0.0297486	0.09693
hsa-CCT2_0004	circCCT2(7,8).1	CCT2	3.29	-1.64	0.75	-2.18	0.0294332	0.09693
hsa-HP1BP3_0004	circHP1BP3(L6,7,8S,9).1	HP1BP3	8.16	-1.04	0.48	-2.16	0.0307456	0.09907

baseMean: Average expression level across all samples. log2FoldChange: Log2-transformed fold change between two conditions, Negative value means downregulated in COVID non-survival and positive means upregulated in COVID non-survival . lfcSE: log2 fold change of standard error. Stat: Statistical test value for differential expression. pvalue: Raw p-value from the statistical test. padj: Adjusted p-value (corrected for multiple testing).

Based on the ≥2 BSJ count matrix, included for transparency. Primary conclusions rely on the two-time-point, precision-weighted Early–Late analysis
