# Supplementary material for: circRNA Signatures Distinguishing COVID-19 Outcomes and Acute Respiratory Distress Syndrome: A Longitudinal, Two-Timepoint, Precision-Weighted Analysis of a Public RNA-Seq Cohort
Source: Genes (Basel). 2025 Dec 30;17(1):34. doi: 10.3390/genes17010034 (PMC12841326; doi:10.3390/genes17010034)
Supplement: Supplementary file 1 [file genes-17-00034-s001.zip › Table S6 Top differentially expressed circRNAs between COVID non-survival and COVID survival at early (Day 3) stage.pdf]

Table S6: Top differentially expressed circRNAs between COVID non-survival and COVID survival at early (Day 3) stage

circAtlas ID	Uniform ID	Gene name	baseMean	Log2Fold Change	lfcSE	Stat	pvalue	padj
hsa-ANKRD12_0008	circANKRD12(S8).1	ANKRD12	27.03	-2.08	0.57	-3.65	0.0003	0.02
hsa-CNST_0007	circCNST(2).1	CNST	3.21	-5.04	1.36	-3.70	0.0002	0.02
hsa-TMEM165_0001	circTMEM165(2,3,4).1	TMEM165	4.67	-4.82	1.24	-3.88	0.0001	0.02
hsa-UBQLN1_0006	circUBQLN1(2,3,4,5).1	UBQLN1	4.22	-4.49	1.18	-3.82	0.0001	0.02
hsa-ERBIN_0001	circERBIN(2,3,4).1	ERBIN	2.71	-4.31	1.32	-3.26	0.0011	0.08
hsa-CBL_0005	circCBL(RI,10,11).1	CBL	2.24	-4.29	1.35	-3.18	0.0015	0.09
hsa-MAN2A1_0001	circMAN2A1(2,3,4).1	MAN2A1	6.38	-2.67	0.86	-3.11	0.0019	0.09

baseMean: Average expression level across all samples. log2FoldChange: Log2-transformed fold change between two conditions, Negative value means downregulated in COVID non-survival and positive means upregulated in COVID non-survival . lfcSE: log2 fold change of standard error. Stat: Statistical test value for differential expression. pvalue: Raw p-value from the statistical test. padj: Adjusted p-value (corrected for multiple testing).

Based on the ≥2 BSJ count matrix, included for transparency. Primary conclusions rely on the two-time-point, precision-weighted Early–Late analysis
