# Supplementary material for: circRNA Signatures Distinguishing COVID-19 Outcomes and Acute Respiratory Distress Syndrome: A Longitudinal, Two-Timepoint, Precision-Weighted Analysis of a Public RNA-Seq Cohort
Source: Genes (Basel). 2025 Dec 30;17(1):34. doi: 10.3390/genes17010034 (PMC12841326; doi:10.3390/genes17010034)
Supplement: Supplementary file 1 [file genes-17-00034-s001.zip › Table S7 Top differentially expressed circRNAs between COVID non-survival and COVID survival at late (Day 7+10) stage.pdf]

Table S7: Top differentially expressed circRNAs between COVID non-survival and COVID survival at late (Day 7+10) stage

circAtlas ID	Uniform ID	Gene name	baseMean	Log2Fold Change	lfcSE	Stat	pvalue	padj
hsa-TMCC2_0001	circTMCC2(3).1	TMCC2	25.48	6.43	1.33	4.84	0.000001	0.0004
hsa-TFRC_0004	circTFRC(2,3,L4,5,6,7,8,9).1	TFRC	17.89	5.57	1.32	4.21	0.000026	0.0037
hsa-SLC14A1_0001	circSLC14A1(7,L8).1	SLC14A1	7.94	4.24	1.13	3.76	0.000172	0.0166
hsa-VMP1_0001	circVMP1(2,3,4,5).1	VMP1	8.17	-1.55	0.43	-3.58	0.000338	0.0190
hsa-ANKRD36BP2	circ(chr2)	ANKRD36BP2	10.80	3.13	0.88	3.54	0.000394	0.0190
hsa-WWC3_0004	circ(chrX).12	WWC3	5.64	4.03	1.13	3.55	0.000382	0.0190
hsa-NUP54_0005	circNUP54(2,3).1	NUP54	3.03	3.77	1.10	3.44	0.000592	0.0245
hsa-SOX6_0034	circSOX6(8,9,10,L11,12,13).1	SOX6	4.14	3.90	1.15	3.39	0.000701	0.0254
hsa-CDYL_0005	circCDYL(2).1	CDYL	60.55	1.62	0.48	3.35	0.000814	0.0262
hsa-SPECC1_0001	circSPECC1(4).1	SPECC1	99.40	2.04	0.63	3.25	0.001140	0.0331
hsa-RHBDD1_0003	circRHBDD1(4,5,6,7,8).1	RHBDD1	10.59	2.41	0.77	3.14	0.001663	0.0402
hsa-SLC36A1_0002	circSLC36A1(2,3,4,5,6,7,8,9,10).1	SLC36A1	4.43	3.76	1.19	3.16	0.001601	0.0402
hsa-RAB6A_0007	circRAB6A(4,6).1	RAB6A	2.48	-2.81	0.91	-3.09	0.001970	0.0415
hsa-RARS_0012	circRARS1(2,3,4,5).1	RARS1	11.13	1.14	0.37	3.09	0.002001	0.0415
hsa-FECH_0013	circFECH(L2,3,4).1	FECH	2.50	3.14	1.04	3.01	0.002596	0.0502
hsa-EIF4E3_0001	circEIF4E3(3,L4,5,6).1	EIF4E3	4.41	-1.76	0.60	-2.95	0.003209	0.0547
hsa-AFF1_0001	circAFF1(3,4).1	AFF1	23.46	1.63	0.55	2.96	0.003056	0.0547
circFOXO3	circFOXO3	FOXO3	5.20	3.03	1.06	2.85	0.004374	0.0668
hsa-AC090094_0001	circASPH(2,3).1	ASPH	18.72	-1.45	0.50	-2.87	0.004162	0.0668
hsa-CLEC16A_0001	circCLEC16A(12,13,14,15,16,17,18,19,20,21,22).1	CLEC16A	8.08	1.57	0.55	2.83	0.004680	0.0679
hsa-TOP1_0001	circTOP1(9,10,11,12,13).1	TOP1	6.48	2.16	0.79	2.74	0.006161	0.0791
hsa-FCHO2_0068	circFCHO2(17,18,19S,20,L21).1	FCHO2	5.12	3.03	1.10	2.75	0.005896	0.0791
hsa-ISPD_0004	circCRPPA(6,7,8S).1	CRPPA	3.09	3.35	1.22	2.73	0.006270	0.0791
hsa-RAB3IP_0001	circRAB3IP(2,3).1	RAB3IP	4.13	2.57	0.95	2.71	0.006630	0.0801

baseMean: Average expression level across all samples. log2FoldChange: Log2-transformed fold change between two conditions, Negative value means downregulated in COVID non-survival and positive means upregulated in COVID non-survival . lfcSE: log2 fold change of standard error. Stat: Statistical test value for differential expression. pvalue: Raw p-value from the statistical test. padj: Adjusted p-value (corrected for multiple testing).

Based on the ≥2 BSJ count matrix, included for transparency. Primary conclusions rely on the two-time-point, precision-weighted Early–Late analysis
