# Supplementary material for: circRNA Signatures Distinguishing COVID-19 Outcomes and Acute Respiratory Distress Syndrome: A Longitudinal, Two-Timepoint, Precision-Weighted Analysis of a Public RNA-Seq Cohort
Source: Genes (Basel). 2025 Dec 30;17(1):34. doi: 10.3390/genes17010034 (PMC12841326; doi:10.3390/genes17010034)
Supplement: Supplementary file 1 [file genes-17-00034-s001.zip › Table S8 Time-Resolved circRNA Differences (Early + Late) - COVID Non-Survivor vs ARDS.pdf]

Table S8:Time-Resolved circRNA Differences (Early + Late) - COVID Non-Survivor vs ARDS

circAtlas ID	Uniform ID	log2FC 95% CI (Combined)*	Adjusted P (Combined)*	Direction	log2FC 95% CI (Early)	log2FC 95% CI (Late)	baseMean (Day3)	baseMean (Late)
hsa-ANKRD12_0008	circANKRD12(S8).1	-2.25 (95% CI -3.00 to -1.50)	0.000001	↑ ARDS	-2.81 (95% CI -3.92 to -1.70)	-1.78 (95% CI -2.79 to -0.76)	27.03	23.06
hsa-PCMTD1_0002	circPCMTD1(2).1	-1.81 (95% CI -2.47 to -1.15)	0.00001	↑ ARDS	-1.82 (95% CI -2.85 to -0.78)	-1.81 (95% CI -2.67 to -0.95)	23.33	26.84
hsa-UBQLN1_0006	circUBQLN1(2,3,4,5).1	-3.38 (95% CI -4.72 to -2.05)	0.0001	↑ ARDS	-4.96 (95% CI -7.25 to -2.67)	-2.57 (95% CI -4.22 to -0.92)	4.22	3.30
hsa-TFRC_0013	circTFRC(3,4).1	-2.44 (95% CI -3.53 to -1.36)	0.001	↑ ARDS	-0.95 (95% CI -3.57 to +1.67)	-2.75 (95% CI -3.94 to -1.56)	3.62	4.18
hsa-NRIP1_0002	circNRIP1(2,3).1	+2.31 (95% CI +1.26 to +3.37)	0.001	↑ COVID (NS)	+1.86 (95% CI +0.10 to +3.61)	+2.57 (95% CI +1.25 to +3.90)	12.98	5.77
hsa-PCNT_0003	circPCNT(7,8).1	-2.27 (95% CI -3.30 to -1.24)	0.001	↑ ARDS	-2.47 (95% CI -4.50 to -0.43)	-2.20 (95% CI -3.40 to -1.01)	5.92	7.58
hsa-DNAJC6_0001	circDNAJC6(2,3,4).1	-2.23 (95% CI -3.29 to -1.17)	0.001	↑ ARDS	-2.06 (95% CI -4.03 to -0.10)	-2.30 (95% CI -3.55 to -1.04)	9.19	9.90
hsa-ZNF516_0005	circZNF516(S3).1	+1.72 (95% CI +0.89 to +2.54)	0.002	↑ COVID (NS)	+2.03 (95% CI +0.76 to +3.30)	+1.48 (95% CI +0.39 to +2.57)	11.26	7.78
hsa-VRK2_0001	circVRK2(3,4,5,6,7).1	-2.51 (95% CI -3.79 to -1.24)	0.002	↑ ARDS	-3.50 (95% CI -6.00 to -1.00)	-2.17 (95% CI -3.65 to -0.69)	3.80	3.13
hsa-ASAP1_0002	circASAP1(8,9,10,11,12,13).1	-2.20 (95% CI -3.32 to -1.09)	0.002	↑ ARDS	-2.71 (95% CI -4.55 to -0.87)	-1.91 (95% CI -3.32 to -0.51)	8.25	8.45
hsa-XPO1_0001	circXPO1(2,3,4).1	-1.41 (95% CI -2.13 to -0.69)	0.002	↑ ARDS	-2.06 (95% CI -3.32 to -0.81)	-1.10 (95% CI -1.97 to -0.23)	19.33	18.27
hsa-RBM33_0009	circRBM33(3,4,5).1	-1.10 (95% CI -1.66 to -0.55)	0.002	↑ ARDS	-1.10 (95% CI -2.21 to +0.00)	-1.10 (95% CI -1.74 to -0.47)	19.85	22.08
hsa-SLTM_0001	circSLTM(L3,L4,5).1	-0.89 (95% CI -1.33 to -0.44)	0.002	↑ ARDS	-1.07 (95% CI -1.98 to -0.17)	-0.83 (95% CI -1.34 to -0.32)	31.71	34.26
hsa-FAM13B_0019	circFAM13B(8,9,10).1	-1.22 (95% CI -1.85 to -0.59)	0.003	↑ ARDS	-2.10 (95% CI -3.26 to -0.93)	-0.86 (95% CI -1.61 to -0.11)	18.51	18.78
hsa-TBCEL_0004	circTBCEL(3,4,5S,6,7S,8).1	-1.51 (95% CI -2.30 to -0.72)	0.003	↑ ARDS	-2.23 (95% CI -3.77 to -0.69)	-1.25 (95% CI -2.17 to -0.33)	4.97	5.33
hsa-RAB6A_0007	circRAB6A(4,6).1	-2.60 (95% CI -4.00 to -1.19)	0.004	↑ ARDS	-1.63 (95% CI -4.23 to +0.97)	-3.00 (95% CI -4.66 to -1.33)	3.20	2.48

hsa-RANBP9_0002	circRANBP9(6,7,8,9).1	-2.57 (95% CI -3.96 to -1.17)	0.004	↑ ARDS	-3.73 (95% CI -6.19 to -1.27)	-2.02 (95% CI -3.71 to -0.33)	3.91	3.02
hsa-PICALM_0007	circPICALM(2,3,4,5,6,L7,8S).1	-2.25 (95% CI -3.47 to -1.03)	0.004	↑ ARDS	-2.82 (95% CI -5.70 to +0.06)	-2.12 (95% CI -3.47 to -0.77)	4.54	4.24
hsa-PICALM_0008	circPICALM(2,3,4,5,6,7).1	-2.22 (95% CI -3.42 to -1.02)	0.004	↑ ARDS	-3.86 (95% CI -6.45 to -1.27)	-1.77 (95% CI -3.13 to -0.41)	3.70	3.96
hsa-SUCO_0003	circSUCO(2,3,L4,5,6).1	-2.10 (95% CI -3.22 to -0.97)	0.004	↑ ARDS	-2.79 (95% CI -4.93 to -0.65)	-1.83 (95% CI -3.15 to -0.51)	6.55	6.63
hsa-GMIP_0001	circGMIP(5,RI,6,7).1	+1.09 (95% CI +0.50 to +1.68)	0.004	↑ COVID (NS)	+1.06 (95% CI +0.08 to +2.04)	+1.11 (95% CI +0.37 to +1.85)	12.14	11.07
hsa-FCHO2_0068	circFCHO2(17,18,19S,20,L21).1	-2.21 (95% CI -3.41 to -1.00)	0.004	↑ ARDS	-3.43 (95% CI -6.12 to -0.73)	-1.90 (95% CI -3.25 to -0.56)	4.49	5.12
hsa-SCARF1_0001	circSCARF1(9,RI,10).1	+1.70 (95% CI +0.75 to +2.64)	0.004	↑ COVID (NS)	+2.08 (95% CI +0.36 to +3.81)	+1.53 (95% CI +0.41 to +2.66)	9.64	6.38
hsa-SPECC1_0001	circSPECC1(4).1	-1.55 (95% CI -2.41 to -0.69)	0.004	↑ ARDS	-1.90 (95% CI -3.27 to -0.53)	-1.32 (95% CI -2.43 to -0.21)	89.11	99.40
hsa-MAN1A2_0003	circMAN1A2(2,3,4,5).1	-1.06 (95% CI -1.65 to -0.47)	0.004	↑ ARDS	-1.57 (95% CI -2.53 to -0.61)	-0.74 (95% CI -1.49 to +0.01)	26.48	20.27
hsa-METTL3_0002	circMETTL3(2,2,RI,3).1	+0.99 (95% CI +0.44 to +1.55)	0.004	↑ COVID (NS)	+1.64 (95% CI +0.66 to +2.62)	+0.69 (95% CI +0.02 to +1.36)	12.19	10.10
hsa-MINPP1_0001	circMINPP1(2,3,L4).1	-2.69 (95% CI -4.20 to -1.18)	0.005	↑ ARDS	-1.05 (95% CI -3.68 to +1.57)	-3.50 (95% CI -5.35 to -1.66)	2.85	2.40
hsa-SLC45A4_0002	circSLC45A4(2).1	+0.85 (95% CI +0.37 to +1.33)	0.005	↑ COVID (NS)	+0.55 (95% CI -0.45 to +1.56)	+0.93 (95% CI +0.39 to +1.48)	14.35	13.45
hsa-DEF6_0002	circDEF6(RI,4,5).1	+1.38 (95% CI +0.60 to +2.17)	0.005	↑ COVID (NS)	+2.12 (95% CI +0.81 to +3.43)	+0.97 (95% CI -0.02 to +1.95)	9.69	9.96
hsa-ASH2L_0010	circASH2L(6,7,L8,9).1	-1.93 (95% CI -3.03 to -0.82)	0.005	↑ ARDS	-1.71 (95% CI -3.92 to +0.50)	-2.00 (95% CI -3.27 to -0.73)	4.02	2.94
hsa-PICALM_0001	circPICALM(2,3,4,5,6,7,8,9,10,11,12S).1	-1.75 (95% CI -2.75 to -0.75)	0.005	↑ ARDS	-2.74 (95% CI -4.70 to -0.78)	-1.40 (95% CI -2.56 to -0.23)	7.16	4.71
hsa-TMEM56-RWDD3_0004	circTLCD4(2S,3,L4,5).1	-2.23 (95% CI -3.51 to -0.94)	0.005	↑ ARDS	-2.88 (95% CI -5.92 to +0.15)	-2.08 (95% CI -3.50 to -0.66)	19.86	19.36
hsa-RNF10_0004	circRNF10(RI,5,6).1	-0.96 (95% CI -1.51 to -0.40)	0.005	↑ ARDS	-0.40 (95% CI -1.52 to +0.72)	-1.14 (95% CI -1.78 to -0.50)	12.20	15.77

hsa-FBXO9_0002	circFBXO9(L4).1	-1.39 (95% CI -2.20 to -0.57)	0.006	↑ ARDS	-1.19 (95% CI -3.02 to +0.63)	-1.43 (95% CI -2.34 to -0.53)	4.88	5.26
hsa-RHBDD1_0004	circRHBDD1(4,5).1	-1.52 (95% CI -2.42 to -0.63)	0.006	↑ ARDS	-2.27 (95% CI -4.44 to -0.11)	-1.37 (95% CI -2.35 to -0.38)	11.58	12.11
hsa-RNF10_0006	circRNF10(5,6,7).1	-2.04 (95% CI -3.25 to -0.84)	0.007	↑ ARDS	-1.23 (95% CI -3.40 to +0.93)	-2.41 (95% CI -3.87 to -0.95)	3.89	3.15
hsa-ZCCHC6_0015	circTUT7(19,20,21,22,23).1	-1.65 (95% CI -2.64 to -0.66)	0.008	↑ ARDS	-1.89 (95% CI -3.65 to -0.14)	-1.53 (95% CI -2.73 to -0.33)	5.47	4.67
hsa-PTPRA_0004	circPTPRA(4,5).1	-1.50 (95% CI -2.41 to -0.59)	0.008	↑ ARDS	-1.76 (95% CI -3.11 to -0.40)	-1.29 (95% CI -2.51 to -0.07)	7.91	8.13
hsa-CCDC66_0011	circCCDC66(RI,5,6).1	-1.39 (95% CI -2.24 to -0.54)	0.009	↑ ARDS	-1.26 (95% CI -2.87 to +0.34)	-1.44 (95% CI -2.44 to -0.44)	5.50	4.37
hsa-RSRC1_0001	circRSRC1(2,3).1	-1.66 (95% CI -2.69 to -0.64)	0.009	↑ ARDS	-2.65 (95% CI -4.38 to -0.93)	-1.12 (95% CI -2.40 to +0.15)	4.67	3.82
hsa-DYNC1H1_0014	circDYNC1H1(RI,63,64).1	+1.12 (95% CI +0.42 to +1.81)	0.010	↑ COVID (NS)	+1.37 (95% CI +0.33 to +2.42)	+0.92 (95% CI -0.01 to +1.84)	12.20	9.01
chr6:108663454-108664889	chr6:108663454-108664889	-1.90 (95% CI -3.09 to -0.72)	0.010	↑ ARDS	-4.23 (95% CI -6.96 to -1.49)	-1.36 (95% CI -2.68 to -0.05)	4.50	5.20
hsa-ITGAL_0005	circITGAL(5,6).1	+1.47 (95% CI +0.55 to +2.39)	0.011	↑ COVID (NS)	+1.97 (95% CI +0.49 to +3.45)	+1.15 (95% CI -0.03 to +2.33)	6.58	6.25
hsa-SCMH1_0001	circSCMH1(L9,10).1	-1.35 (95% CI -2.21 to -0.49)	0.012	↑ ARDS	-1.46 (95% CI -2.71 to -0.21)	-1.25 (95% CI -2.44 to -0.07)	6.24	4.97
hsa-VRK1_0002	circVRK1(2,3,4,5,6,7,8,9,10,11).1	-1.12 (95% CI -1.83 to -0.41)	0.012	↑ ARDS	-1.31 (95% CI -2.40 to -0.22)	-0.98 (95% CI -1.92 to -0.04)	37.87	31.70
hsa-MAN2A1_0001	circMAN2A1(2,3,4).1	-1.34 (95% CI -2.21 to -0.46)	0.015	↑ ARDS	-2.62 (95% CI -4.31 to -0.94)	-0.87 (95% CI -1.89 to +0.16)	6.38	6.28
hsa-ARHGAP26_0002	circARHGAP26(15,16,17).1	+1.10 (95% CI +0.38 to +1.82)	0.015	↑ COVID (NS)	+1.04 (95% CI -0.38 to +2.47)	+1.12 (95% CI +0.28 to +1.95)	7.73	7.48
hsa-SEC62_0004	circSEC62(3,L4,5,6,7).1	-1.01 (95% CI -1.67 to -0.35)	0.015	↑ ARDS	-0.90 (95% CI -1.84 to +0.04)	-1.12 (95% CI -2.05 to -0.19)	12.61	10.31
hsa-SWT1_0003	circSWT1(14,15,16).1	-2.25 (95% CI -3.73 to -0.77)	0.015	↑ ARDS	-2.90 (95% CI -5.46 to -0.35)	-1.93 (95% CI -3.74 to -0.11)	3.58	3.95
hsa-AKAP7_0001	circAKAP7(2,L3,4,5).1	-1.60 (95% CI -2.65 to -0.54)	0.016	↑ ARDS	-1.19 (95% CI -3.14 to +0.76)	-1.76 (95% CI -3.02 to -0.50)	4.64	5.16
hsa-N4BP2L2_0001	circN4BP2L2(3,4,5S,S6).2	-0.98 (95% CI -1.63 to -0.33)	0.016	↑ ARDS	-1.08 (95% CI -2.18 to +0.02)	-0.93 (95% CI -1.73 to -0.12)	32.32	26.70

hsa-ARHGEF12_0041	circARHGEF12(32,33L,34,35,36).1	-2.33 (95% CI -3.88 to -0.77)	0.016	↑ ARDS	-3.02 (95% CI -5.46 to -0.57)	-1.86 (95% CI -3.87 to +0.15)	4.98	2.52
hsa-ISPD_0004	circCRPPA(6,7,8S).1	-1.98 (95% CI -3.30 to -0.66)	0.016	↑ ARDS	-2.26 (95% CI -4.41 to -0.10)	-1.81 (95% CI -3.49 to -0.13)	3.64	3.09
hsa-SUCO_0022	circSUCO(4,5,6).1	-1.96 (95% CI -3.27 to -0.64)	0.017	↑ ARDS	-2.20 (95% CI -5.01 to +0.62)	-1.89 (95% CI -3.38 to -0.40)	2.75	3.37
hsa-MARCH6_0047	circMARCHF6(21,22).1	+1.22 (95% CI +0.40 to +2.05)	0.017	↑ COVID (NS)	+2.30 (95% CI +0.94 to +3.65)	+0.58 (95% CI -0.46 to +1.63)	8.79	6.23
hsa-ITGAL_0003	circITGAL(RI,14,15).1	+1.03 (95% CI +0.33 to +1.72)	0.017	↑ COVID (NS)	+2.00 (95% CI +0.99 to +3.02)	+0.17 (95% CI -0.78 to +1.12)	15.00	20.13
hsa-CREBBP_0001	circCREBBP(2).	-0.79 (95% CI -1.33 to -0.26)	0.017	↑ ARDS	-0.11 (95% CI -1.16 to +0.95)	-1.03 (95% CI -1.65 to -0.41)	6.86	8.08
hsa-RHBDD1_0003	circRHBDD1(4,5,6,7,8).1	-1.40 (95% CI -2.35 to -0.44)	0.018	↑ ARDS	-1.56 (95% CI -3.50 to +0.39)	-1.35 (95% CI -2.44 to -0.25)	10.37	10.59
hsa-SOX6_0034	circSOX6(8,9,10,L11,12,13).1	-1.94 (95% CI -3.29 to -0.60)	0.020	↑ ARDS	-3.51 (95% CI -6.70 to -0.32)	-1.60 (95% CI -3.08 to -0.12)	3.61	4.14
hsa-ANKRD36BP2	circ(chr2)	-1.42 (95% CI -2.41 to -0.44)	0.020	↑ ARDS	-0.80 (95% CI -2.87 to +1.26)	-1.61 (95% CI -2.73 to -0.48)	6.60	10.80
hsa-IL27RA_0001	circIL27RA(5,6).1	+1.38 (95% CI +0.43 to +2.33)	0.020	↑ COVID (NS)	+2.51 (95% CI +1.09 to +3.93)	+0.46 (95% CI -0.82 to +1.74)	7.53	8.43
hsa-MBOAT2_0001	circMBOAT2(2,3).1	-1.77 (95% CI -3.02 to -0.51)	0.024	↑ ARDS	-1.55 (95% CI -3.74 to +0.65)	-1.88 (95% CI -3.41 to -0.34)	4.38	3.97
hsa-FCHO2_0038	circFCHO2(20,21).1	-1.13 (95% CI -1.94 to -0.32)	0.025	↑ ARDS	-0.40 (95% CI -2.14 to +1.34)	-1.33 (95% CI -2.24 to -0.42)	9.95	8.42
hsa-EP300_0036	circEP300(3,4,5,6,7,8,9).1	-2.09 (95% CI -3.62 to -0.57)	0.029	↑ ARDS	-2.06 (95% CI -4.53 to +0.40)	-2.11 (95% CI -4.05 to -0.16)	3.54	2.84
hsa-EZH2_0001	circEZH2(2,3).1	-1.32 (95% CI -2.29 to -0.36)	0.029	↑ ARDS	-0.87 (95% CI -2.35 to +0.61)	-1.66 (95% CI -2.94 to -0.39)	3.94	3.20
hsa-MIB1_0003	circMIB1(2,3,4,L5,6).1	-1.02 (95% CI -1.77 to -0.27)	0.030	↑ ARDS	-1.29 (95% CI -2.37 to -0.21)	-0.77 (95% CI -1.82 to +0.28)	8.83	8.48
hsa-CTBP1_0001	circCTBP1(2,3,4,5).1	+0.85 (95% CI +0.22 to +1.48)	0.030	↑ COVID (NS)	+1.20 (95% CI +0.09 to +2.30)	+0.69 (95% CI -0.08 to +1.45)	12.11	10.58
hsa-CAPZA1_0004	circCAPZA1(4,5,6,7).1	-1.27 (95% CI -2.22 to -0.32)	0.034	↑ ARDS	-1.01 (95% CI -2.53 to +0.52)	-1.43 (95% CI -2.65 to -0.22)	4.22	3.98
hsa-CCDC66_0010	circCCDC66(8,9,10).1	-1.16 (95% CI -2.03 to -0.29)	0.034	↑ ARDS	-2.15 (95% CI -3.95 to -0.35)	-0.86 (95% CI -1.85 to +0.14)	5.81	6.23
hsa-USP25_0001	circUSP25(2,3).1	-0.96 (95% CI -1.68 to -0.24)	0.034	↑ ARDS	-1.14 (95% CI -2.61 to +0.32)	-0.90 (95% CI -1.73 to -0.07)	7.09	9.76

hsa-SMARCA5_0005	circSMARCA5(15,L16).1	-0.93 (95% CI -1.62 to -0.23)	0.034	↑ ARDS	-1.11 (95% CI -2.27 to +0.04)	-0.82 (95% CI -1.69 to +0.06)	10.60	7.74
hsa-POLD1_0002	circPOLD1(2,L3).1	+1.05 (95% CI +0.25 to +1.84)	0.035	↑ COVID (NS)	+1.47 (95% CI +0.20 to +2.74)	+0.78 (95% CI -0.24 to +1.80)	12.86	12.36
hsa-SCNM1_0001	circSCNM1(3,RI,4,5).1	-0.96 (95% CI -1.69 to -0.23)	0.036	↑ ARDS	-0.64 (95% CI -1.78 to +0.49)	-1.19 (95% CI -2.15 to -0.23)	7.51	6.60
hsa-MED13_0003	circMED13(4,5).1	-1.27 (95% CI -2.26 to -0.29)	0.038	↑ ARDS	-1.49 (95% CI -3.45 to +0.48)	-1.20 (95% CI -2.34 to -0.06)	4.21	3.81
hsa-ZMYND8_0005	circZMYND8(8,L9,10,11).1	-1.04 (95% CI -1.85 to -0.24)	0.038	↑ ARDS	-0.61 (95% CI -2.73 to +1.51)	-1.11 (95% CI -1.98 to -0.24)	3.39	5.37
hsa-FAM188A_0002	circMINDY3(2,3,4,5,6,7,8,9,10).1	-0.90 (95% CI -1.59 to -0.20)	0.038	↑ ARDS	-0.92 (95% CI -2.16 to +0.31)	-0.88 (95% CI -1.72 to -0.05)	5.20	5.60
hsa-CCDC91_0048	circCCDC91(L2,3).2	-0.92 (95% CI -1.64 to -0.20)	0.039	↑ ARDS	-0.64 (95% CI -1.66 to +0.38)	-1.20 (95% CI -2.21 to -0.19)	7.43	6.75
hsa-HERC1_0035	circHERC1(22,23,24,25,26,27).1	-1.20 (95% CI -2.15 to -0.25)	0.043	↑ ARDS	-1.38 (95% CI -3.09 to +0.32)	-1.12 (95% CI -2.26 to +0.02)	8.69	9.51
hsa-UBR2_0001	circUBR2(5,6,7).1	-1.51 (95% CI -2.70 to -0.31)	0.044	↑ ARDS	-0.82 (95% CI -2.52 to +0.89)	-2.18 (95% CI -3.86 to -0.50)	3.71	2.44
hsa-ANKRD17_0008	circANKRD17(29).1	-1.72 (95% CI -3.08 to -0.35)	0.044	↑ ARDS	-2.38 (95% CI -4.73 to -0.03)	-1.38 (95% CI -3.06 to +0.31)	3.45	3.05
hsa-FBXW7_0005	circFBXW7(3,4).1	-0.51 (95% CI -0.91 to -0.10)	0.044	↑ ARDS	-0.81 (95% CI -1.46 to -0.16)	-0.32 (95% CI -0.83 to +0.20)	43.41	39.44
hsa-AURKA_0004	circAURKA(4,5,6).1	-1.37 (95% CI -2.47 to -0.27)	0.046	↑ ARDS	-1.59 (95% CI -3.98 to +0.81)	-1.31 (95% CI -2.56 to -0.07)	3.59	3.49
hsa-SHKBP1_0002	circSHKBP1(RI,11,12).1	+0.75 (95% CI +0.15 to +1.35)	0.046	↑ COVID (NS)	+0.62 (95% CI -0.41 to +1.65)	+0.81 (95% CI +0.08 to +1.55)	12.67	7.72
hsa-CLASP2_0011	circCLASP2(2,3,4,5,6).1	-0.74 (95% CI -1.34 to -0.14)	0.047	↑ ARDS	-1.48 (95% CI -2.73 to -0.23)	-0.52 (95% CI -1.21 to +0.16)	8.36	7.72
hsa-IFI30_0001	circIFI30(2,3,4).1	+0.75 (95% CI +0.14 to +1.35)	0.047	↑ COVID (NS)	+1.30 (95% CI +0.14 to +2.47)	+0.54 (95% CI -0.17 to +1.25)	13.44	14.17
hsa-EP300_0003	circEP300(7,8,9).1	-1.37 (95% CI -2.48 to -0.25)	0.049	↑ ARDS	-2.66 (95% CI -4.97 to -0.35)	-0.97 (95% CI -2.25 to +0.30)	3.63	4.45
hsa-NFATC3_0001	circNFATC3(2,3).1	-0.68 (95% CI -1.25 to -0.11)	0.056	↑ ARDS	-1.16 (95% CI -1.98 to -0.35)	-0.22 (95% CI -1.02 to +0.57)	12.55	11.78
hsa-RAB3D_0003	circRAB3D(3,4).1	+1.05 (95% CI +0.17 to +1.92)	0.056	↑ COVID (NS)	+1.32 (95% CI +0.15 to +2.48)	+0.69 (95% CI -0.64 to +2.03)	8.36	7.53

hsa-CCSER2_0002	circCCSER2(4,5).1	+0.92 (95% CI +0.15 to +1.68)	0.056	↑ COVID (NS)	+1.02 (95% CI -0.50 to +2.54)	+0.88 (95% CI -0.01 to +1.77)	7.38	8.04
hsa-MBOAT2_0003	circMBOAT2(2,3,4).1	-0.89 (95% CI -1.64 to -0.14)	0.057	↑ ARDS	-0.79 (95% CI -2.15 to +0.57)	-0.94 (95% CI -1.84 to -0.04)	13.35	11.08
hsa-GCN1_0003	circGCN1(29,30,31).1	+0.84 (95% CI +0.13 to +1.55)	0.060	↑ COVID (NS)	+2.16 (95% CI +0.63 to +3.68)	+0.47 (95% CI -0.34 to +1.27)	10.78	8.32
hsa-RCL1_0008	circRCL1(2,3).1	-1.36 (95% CI -2.55 to -0.18)	0.068	↑ ARDS	-0.16 (95% CI -2.79 to +2.48)	-1.67 (95% CI -2.99 to -0.34)	4.00	5.33
hsa-FKBP8_0005	circFKBP8(3,4).1	+0.92 (95% CI +0.11 to +1.73)	0.072	↑ COVID (NS)	+1.44 (95% CI +0.16 to +2.73)	+0.57 (95% CI -0.47 to +1.62)	6.65	7.24
hsa-CHSY1_0001	circCHSY1(2).1	+0.78 (95% CI +0.08 to +1.47)	0.077	↑ COVID (NS)	+1.07 (95% CI -0.17 to +2.31)	+0.64 (95% CI -0.19 to +1.48)	7.76	6.88
hsa-RAB3IP_0013	circRAB3IP(7,8).1	-0.96 (95% CI -1.82 to -0.10)	0.078	↑ ARDS	-0.80 (95% CI -2.31 to +0.71)	-1.03 (95% CI -2.08 to +0.01)	4.68	4.37
hsa-TMEM56-RWDD3_0001	circTLCD4(2S,3,4,5,6).1	-1.11 (95% CI -2.11 to -0.10)	0.081	↑ ARDS	-0.19 (95% CI -2.34 to +1.96)	-1.36 (95% CI -2.50 to -0.23)	5.44	6.54
hsa-BPTF_0045	circBPTF(21S,22,23,25,26,27).1	-1.08 (95% CI -2.06 to -0.09)	0.087	↑ ARDS	-1.18 (95% CI -2.64 to +0.27)	-0.99 (95% CI -2.33 to +0.36)	7.83	8.32
hsa-ZFAND6_0001	circZFAND6(3,4,5).1	-0.97 (95% CI -1.86 to -0.07)	0.089	↑ ARDS	-1.86 (95% CI -3.62 to -0.09)	-0.66 (95% CI -1.69 to +0.37)	6.20	5.90
hsa-SETD2_0001	circSETD2(6,7S,8,9S).1	-0.79 (95% CI -1.53 to -0.05)	0.091	↑ ARDS	-0.61 (95% CI -1.85 to +0.62)	-0.89 (95% CI -1.80 to +0.03)	11.06	11.14
hsa-RAB11FIP1_0002	circRAB11FIP1(2).1	+0.71 (95% CI +0.05 to +1.38)	0.091	↑ COVID (NS)	+1.10 (95% CI -0.26 to +2.45)	+0.59 (95% CI -0.17 to +1.36)	6.01	9.07
hsa-CSNK1G3_0001	circCSNK1G3(2,3,4).1	-0.70 (95% CI -1.36 to -0.04)	0.093	↑ ARDS	-1.47 (95% CI -2.64 to -0.30)	-0.35 (95% CI -1.14 to +0.45)	15.41	13.56
hsa-MYO9B_0005	circMYO9B(2).1	+0.79 (95% CI +0.05 to +1.53)	0.094	↑ COVID (NS)	+1.99 (95% CI +0.57 to +3.40)	+0.33 (95% CI -0.54 to +1.20)	14.59	9.95
hsa-ABHD2_0002	circABHD2(2,3).1	-0.55 (95% CI -1.06 to -0.03)	0.095	↑ ARDS	-0.79 (95% CI -1.77 to +0.18)	-0.45 (95% CI -1.06 to +0.16)	12.61	17.52
hsa-ERBIN_0001	circERBIN(2,3,4).1	-1.38 (95% CI -2.70 to -0.06)	0.098	↑ ARDS	-3.91 (95% CI -6.52 to -1.30)	-0.52 (95% CI -2.04 to +1.01)	2.71	3.18
hsa-DDI2_0008	circDDI2(5,6).1	-1.35 (95% CI -2.65 to -0.06)	0.099	↑ ARDS	-1.00 (95% CI -3.90 to +1.91)	-1.44 (95% CI -2.89 to +0.00)	2.76	3.41

*Combined (Day 3 + Late) effects were estimated by inverse-variance–weighted fixed-effects meta-analysis; two-sided p values were FDR-adjusted (Benjamini–Hochberg). (NS)=non-survival.
