# Supplementary material for: circRNA Signatures Distinguishing COVID-19 Outcomes and Acute Respiratory Distress Syndrome: A Longitudinal, Two-Timepoint, Precision-Weighted Analysis of a Public RNA-Seq Cohort
Source: Genes (Basel). 2025 Dec 30;17(1):34. doi: 10.3390/genes17010034 (PMC12841326; doi:10.3390/genes17010034)
Supplement: Supplementary file 1 [file genes-17-00034-s001.zip › Table S9 Time-Resolved circRNA Differences (Early+ Late) - COVID Survivor vs ARDS.pdf]

Table S9 Time-Resolved circRNA Differences (Day 3 + Late) - COVID Survivor vs ARDS

circAtlas ID	Uniform ID	log2FC 95% CI (Combined)*	Adjusted P (Combined)*	Direction	log2FC 95% CI (Early)	log2FC 95% CI (Late)	baseMean (Day3)	baseMean (Late)
hsa-SPECC1_0001	circSPECC1(4).1	-2.66 (95% CI -3.61 to -1.72)	0.00001	↑ ARDS	-1.75 (95% CI -3.18 to -0.31)	-3.36 (95% CI -4.62 to -2.11)	89.11	99.40
hsa-ANKRD36BP2	circ(chr2)	-3.60 (95% CI -4.95 to -2.25)	0.00002	↑ ARDS	-1.73 (95% CI -3.93 to +0.46)	-4.74 (95% CI -6.45 to -3.02)	6.60	10.80
hsa-FCHO2_0068	circFCHO2(17,18,19S,20,L21).1	-4.03 (95% CI -5.66 to -2.40)	0.0001	↑ ARDS	-2.67 (95% CI -5.25 to -0.09)	-4.93 (95% CI -7.04 to -2.83)	4.49	5.12
hsa-TMCC2_0001	circTMCC2(3).1	-3.22 (95% CI -4.52 to -1.93)	0.0001	↑ ARDS	-2.16 (95% CI -3.64 to -0.67)	-6.58 (95% CI -9.22 to -3.94)	20.10	25.48
hsa-SOX6_0034	circSOX6(8,9,10,L11,12,13).1	-4.28 (95% CI -6.05 to -2.50)	0.0001	↑ ARDS	-2.02 (95% CI -5.02 to +0.98)	-5.50 (95% CI -7.71 to -3.29)	3.61	4.14
hsa-RHBDD1_0003	circRHBDD1(4,5,6,7,8).1	-2.83 (95% CI -4.02 to -1.64)	0.0001	↑ ARDS	-1.17 (95% CI -3.16 to +0.83)	-3.76 (95% CI -5.25 to -2.27)	10.37	10.59
hsa-RHBDD1_0004	circRHBDD1(4,5).1	-2.46 (95% CI -3.53 to -1.40)	0.0002	↑ ARDS	-1.07 (95% CI -3.26 to +1.12)	-2.89 (95% CI -4.11 to -1.67)	11.58	12.11
hsa-PCNT_0003	circPCNT(7,8).1	-2.80 (95% CI -4.03 to -1.56)	0.0003	↑ ARDS	-1.19 (95% CI -3.11 to +0.74)	-3.93 (95% CI -5.54 to -2.31)	5.92	7.58
hsa-NRIP1_0002	circNRIP1(2,3).1	+2.57 (95% CI +1.44 to +3.70)	0.0003	↑ COVID (S)	+3.70 (95% CI +1.94 to +5.46)	+1.77 (95% CI +0.29 to +3.24)	12.98	5.77
hsa-TFRC_0013	circTFRC(3,4).1	-3.02 (95% CI -4.37 to -1.67)	0.0003	↑ ARDS	-1.32 (95% CI -4.05 to +1.40)	-3.57 (95% CI -5.12 to -2.02)	3.62	4.18
hsa-ZMYND8_0005	circZMYND8(8,L9,10,11).1	-2.41 (95% CI -3.49 to -1.32)	0.0003	↑ ARDS	-1.43 (95% CI -3.68 to +0.82)	-2.70 (95% CI -3.94 to -1.47)	3.39	5.37
hsa-HERC1_0035	circHERC1(22,23,24,25,26,27).1	-2.37 (95% CI -3.47 to -1.27)	0.0005	↑ ARDS	-1.78 (95% CI -3.56 to -0.01)	-2.74 (95% CI -4.14 to -1.33)	8.69	9.51
hsa-AFF1_0001	circAFF1(3,4).1	-1.67 (95% CI -2.44 to -0.89)	0.0005	↑ ARDS	-1.22 (95% CI -2.32 to -0.13)	-2.10 (95% CI -3.19 to -1.01)	18.66	23.46
chr6:108663454-108664889	chr6:108663454-108664889	-3.18 (95% CI -4.71 to -1.64)	0.001	↑ ARDS	-1.65 (95% CI -3.95 to +0.66)	-4.40 (95% CI -6.45 to -2.34)	4.50	5.20
hsa-RNF10_0004	circRNF10(RI,5,6).1	-1.26 (95% CI -1.88 to -0.65)	0.001	↑ ARDS	-0.83 (95% CI -1.99 to +0.34)	-1.43 (95% CI -2.16 to -0.71)	12.20	15.77
hsa-TMEM56-RWDD3_0004	circTLCD4(2S,3,L4,5).1	-2.97 (95% CI -4.43 to -1.52)	0.001	↑ ARDS	-1.37 (95% CI -4.51 to +1.77)	-3.42 (95% CI -5.06 to -1.77)	19.86	19.36
hsa-MINPP1_0001	circMINPP1(2,3,L4).1	-3.43 (95% CI -5.13 to	0.001	↑ ARDS	-1.04 (95% CI -3.72 to	-5.05 (95% CI -7.25 to	2.85	2.40

		-1.73)			+1.64)	-2.85)		
hsa-WWC3_0004	circ(chrX).12	-2.63 (95% CI -3.93 to -1.33)	0.001	↑ ARDS	-1.61 (95% CI -3.21 to -0.01)	-4.58 (95% CI -6.80 to -2.36)	5.92	5.64
hsa-ARHGAP26_0002	circARHGAP26(15,16,17).1	+1.50 (95% CI +0.76 to +2.25)	0.001	↑ COVID (S)	+0.47 (95% CI -1.01 to +1.95)	+1.85 (95% CI +0.99 to +2.72)	7.73	7.48
hsa-TFRC_0004	circTFRC(2,3,L4,5,6,7,8,9).1	-3.41 (95% CI -5.11 to -1.71)	0.001	↑ ARDS	-1.25 (95% CI -3.49 to +0.98)	-6.38 (95% CI -9.00 to -3.75)	12.50	17.89
hsa-RAB11FIP1_0002	circRAB11FIP1(2).1	+1.36 (95% CI +0.68 to +2.04)	0.001	↑ COVID (S)	+1.20 (95% CI -0.15 to +2.55)	+1.41 (95% CI +0.63 to +2.20)	6.01	9.07
hsa-TBCEL_0004	circTBCEL(3,4,5S,6,7S,8).1	-1.68 (95% CI -2.54 to -0.82)	0.001	↑ ARDS	-1.40 (95% CI -2.77 to -0.03)	-1.87 (95% CI -2.97 to -0.76)	4.97	5.33
hsa-SLC14A1_0001	circSLC14A1(7,L8).1	-3.00 (95% CI -4.56 to -1.45)	0.002	↑ ARDS	-1.44 (95% CI -3.61 to +0.74)	-4.64 (95% CI -6.86 to -2.41)	6.35	7.94
hsa-VRK1_0002	circVRK1(2,3,4,5,6,7,8,9,10,11).1	-1.46 (95% CI -2.24 to -0.69)	0.002	↑ ARDS	-0.94 (95% CI -2.06 to +0.18)	-1.94 (95% CI -3.01 to -0.87)	37.87	31.70
hsa-DNAJC6_0001	circDNAJC6(2,3,4).1	-2.20 (95% CI -3.38 to -1.01)	0.003	↑ ARDS	-0.34 (95% CI -2.26 to +1.58)	-3.33 (95% CI -4.84 to -1.83)	9.19	9.90
hsa-EZH2_0001	circEZH2(2,3).1	-2.12 (95% CI -3.28 to -0.96)	0.003	↑ ARDS	-1.26 (95% CI -2.77 to +0.25)	-3.37 (95% CI -5.19 to -1.55)	3.94	3.20
hsa-AC090094_0001	circASPH(2,3).1	+1.49 (95% CI +0.67 to +2.31)	0.003	↑ COVID (S)	+0.81 (95% CI -0.59 to +2.21)	+1.84 (95% CI +0.84 to +2.84)	22.27	18.72
hsa-RCL1_0008	circRCL1(2,3).1	-2.83 (95% CI -4.40 to -1.26)	0.004	↑ ARDS	-0.35 (95% CI -3.09 to +2.39)	-4.05 (95% CI -5.97 to -2.13)	4.00	5.33
hsa-CDYL_0005	circCDYL(2).1	-1.26 (95% CI -1.96 to -0.55)	0.004	↑ ARDS	-0.65 (95% CI -1.68 to +0.39)	-1.80 (95% CI -2.76 to -0.83)	48.09	60.55
hsa-GMIP_0001	circGMIP(5,RI,6,7).1	+1.11 (95% CI +0.48 to +1.73)	0.004	↑ COVID (S)	+0.57 (95% CI -0.44 to +1.59)	+1.43 (95% CI +0.64 to +2.22)	12.14	11.07
hsa-ZNF516_0005	circZNF516(S3).1	+1.55 (95% CI +0.67 to +2.43)	0.005	↑ COVID (S)	+0.78 (95% CI -0.57 to +2.12)	+2.13 (95% CI +0.97 to +3.29)	11.26	7.78
hsa-EPB41_0021	circEPB41(10,11).1	-2.28 (95% CI -3.59 to -0.97)	0.005	↑ ARDS	-1.31 (95% CI -3.65 to +1.04)	-2.72 (95% CI -4.29 to -1.14)	4.15	3.99
hsa-ISPD_0004	circCRPPA(6,7,8S).1	-2.58 (95% CI -4.07 to -1.09)	0.005	↑ ARDS	-0.79 (95% CI -2.73 to +1.14)	-5.16 (95% CI -7.49 to -2.83)	3.64	3.09
hsa-RNF10_0006	circRNF10(5,6,7).1	-2.33 (95% CI -3.68 to -0.98)	0.005	↑ ARDS	-1.69 (95% CI -3.94 to +0.56)	-2.69 (95% CI -4.38 to -1.00)	3.89	3.15
hsa-ARHGEF12_0041	circARHGEF12(32,33L,34,35,36).1	-2.93 (95% CI -4.64 to -1.22)	0.005	↑ ARDS	-1.43 (95% CI -3.70 to +0.84)	-4.89 (95% CI -7.49 to -2.29)	4.98	2.52

hsa-MYO9B_0005	circMYO9B(2).1	+1.34 (95% CI +0.56 to +2.11)	0.005	↑ COVID (S)	+1.62 (95% CI +0.14 to +3.09)	+1.23 (95% CI +0.31 to +2.14)	14.59	9.95
hsa-RBM33_0009	circRBM33(3,4,5).1	-1.03 (95% CI -1.63 to -0.43)	0.005	↑ ARDS	-0.40 (95% CI -1.51 to +0.71)	-1.29 (95% CI -2.01 to -0.58)	19.85	22.08
hsa-AKAP7_0001	circAKAP7(2,L3,4,5).1	-2.08 (95% CI -3.31 to -0.85)	0.006	↑ ARDS	-0.80 (95% CI -2.75 to +1.16)	-2.93 (95% CI -4.51 to -1.34)	4.64	5.16
hsa-TOP1_0001	circTOP1(9,10,11,12,13).1	-1.73 (95% CI -2.75 to -0.71)	0.006	↑ ARDS	-1.28 (95% CI -2.63 to +0.07)	-2.33 (95% CI -3.88 to -0.78)	10.13	6.48
hsa-CREBBP_0001	circCREBBP(2).	-0.98 (95% CI -1.56 to -0.40)	0.006	↑ ARDS	-1.27 (95% CI -2.43 to -0.11)	-0.89 (95% CI -1.56 to -0.22)	6.86	8.08
hsa-DEF6_0002	circDEF6(RI,4,5).1	+1.41 (95% CI +0.58 to +2.25)	0.006	↑ COVID (S)	+1.83 (95% CI +0.49 to +3.18)	+1.15 (95% CI +0.07 to +2.22)	9.69	9.96
hsa-PCMTD1_0002	circPCMTD1(2).1	-1.16 (95% CI -1.85 to -0.47)	0.006	↑ ARDS	-0.70 (95% CI -1.72 to +0.31)	-1.56 (95% CI -2.51 to -0.62)	23.33	26.84
hsa-FKBP8_0002	circFKBP8(L5,6).1	-1.35 (95% CI -2.16 to -0.53)	0.007	↑ ARDS	-1.44 (95% CI -2.58 to -0.31)	-1.25 (95% CI -2.42 to -0.08)	8.42	7.28
hsa-VMP1_0001	circVMP1(2,3,4,5).1	+1.20 (95% CI +0.47 to +1.92)	0.007	↑ COVID (S)	+0.19 (95% CI -1.44 to +1.82)	+1.44 (95% CI +0.63 to +2.25)	5.39	8.17
hsa-SOX6_0009	circSOX6(5,6).1	-3.08 (95% CI -4.96 to -1.21)	0.007	↑ ARDS	-1.61 (95% CI -4.93 to +1.72)	-3.78 (95% CI -6.05 to -1.50)	5.10	3.63
hsa-ARHGEF12_0042	circARHGEF12(31,32,33L,34,35,36).1	-3.18 (95% CI -5.14 to -1.23)	0.008	↑ ARDS	-2.16 (95% CI -5.63 to +1.32)	-3.66 (95% CI -6.02 to -1.30)	4.12	3.10
hsa-DDI2_0008	circDDI2(5,6).1	-2.77 (95% CI -4.49 to -1.06)	0.008	↑ ARDS	-0.72 (95% CI -3.67 to +2.24)	-3.82 (95% CI -5.93 to -1.71)	2.76	3.41
hsa-SLC37A3_0005	circSLC37A3(6,7).1	+1.46 (95% CI +0.53 to +2.39)	0.012	↑ COVID (S)	+1.58 (95% CI -0.54 to +3.70)	+1.43 (95% CI +0.39 to +2.47)	7.97	9.05
hsa-CLEC16A_0001	circCLEC16A(12,13,14,15,16,17,18,19,20,21,22).1	-1.37 (95% CI -2.24 to -0.49)	0.012	↑ ARDS	-1.31 (95% CI -2.79 to +0.17)	-1.40 (95% CI -2.49 to -0.31)	5.70	8.08
hsa-MVP_0002	circMVP(4,5).1	+1.13 (95% CI +0.38 to +1.89)	0.017	↑ COVID (S)	+1.49 (95% CI -0.19 to +3.16)	+1.04 (95% CI +0.20 to +1.89)	9.90	9.79
hsa-SEC62_0004	circSEC62(3,L4,5,6,7).1	-1.04 (95% CI -1.74 to -0.35)	0.017	↑ ARDS	-0.65 (95% CI -1.57 to +0.27)	-1.56 (95% CI -2.62 to -0.50)	12.61	10.31
hsa-PICALM_0001	circPICALM(2,3,4,5,6,7,8,9,10,11,12S).1	-1.58 (95% CI -2.66 to -0.51)	0.019	↑ ARDS	-1.53 (95% CI -3.40 to +0.33)	-1.61 (95% CI -2.92 to -0.29)	7.16	4.71
hsa-SWT1_0003	circSWT1(14,15,16).1	-2.34 (95% CI -3.93 to -0.75)	0.019	↑ ARDS	-1.74 (95% CI -4.11 to +0.63)	-2.83 (95% CI -4.97 to -0.68)	3.58	3.95
hsa-NCAPG_0005	circNCAPG(4,5).1	-2.10 (95% CI -3.53 to -0.67)	0.019	↑ ARDS	-0.82 (95% CI -3.03 to +1.40)	-3.01 (95% CI -4.89 to -1.14)	4.28	4.30

hsa-SLC45A4_0002	circSLC45A4(2).1	+0.76 (95% CI +0.24 to +1.27)	0.019	↑ COVID (S)	+0.39 (95% CI -0.64 to +1.42)	+0.88 (95% CI +0.28 to +1.47)	14.35	13.45
hsa-TMEM56-RWDD3_0001	circTLCD4(2S,3,4,5,6).1	-1.68 (95% CI -2.84 to -0.53)	0.020	↑ ARDS	-0.07 (95% CI -2.28 to +2.15)	-2.29 (95% CI -3.65 to -0.93)	5.44	6.54
hsa-NAP1L4_0006	circNAP1L4(2,3,4,5S,6,7S,8,9,11,12,13,14).1	-2.57 (95% CI -4.35 to -0.79)	0.021	↑ ARDS	-0.61 (95% CI -3.46 to +2.24)	-3.82 (95% CI -6.10 to -1.54)	3.19	5.35
hsa-NPRL3_0003	circNPRL3(L8,9).1	-2.01 (95% CI -3.44 to -0.58)	0.025	↑ ARDS	-0.65 (95% CI -3.06 to +1.75)	-2.75 (95% CI -4.52 to -0.98)	5.21	4.78
hsa-IL27RA_0001	circIL27RA(5,6).1	+1.41 (95% CI +0.40 to +2.43)	0.027	↑ COVID (S)	+1.71 (95% CI +0.23 to +3.19)	+1.15 (95% CI -0.24 to +2.54)	7.53	8.43
hsa-TTC39C_0002	circTTC39C(2,3,4).1	+1.03 (95% CI +0.29 to +1.77)	0.027	↑ COVID (S)	+1.26 (95% CI -0.03 to +2.55)	+0.92 (95% CI +0.02 to +1.83)	6.21	6.07
hsa-PTPRA_0004	circPTPRA(4,5).1	-1.29 (95% CI -2.24 to -0.35)	0.030	↑ ARDS	-1.23 (95% CI -2.54 to +0.08)	-1.36 (95% CI -2.72 to -0.00)	7.91	8.13
hsa-FCHO2_0038	circFCHO2(20,21).1	-1.23 (95% CI -2.13 to -0.34)	0.030	↑ ARDS	-0.16 (95% CI -1.96 to +1.63)	-1.58 (95% CI -2.62 to -0.55)	9.95	8.42
hsa-PLEKHM1_0002	circPLEKHM1(4).1	-0.87 (95% CI -1.51 to -0.23)	0.032	↑ ARDS	-1.63 (95% CI -2.64 to -0.61)	-0.37 (95% CI -1.19 to +0.45)	9.60	7.89
hsa-FBXW7_0005	circFBXW7(3,4).1	-0.59 (95% CI -1.03 to -0.15)	0.032	↑ ARDS	-0.39 (95% CI -1.04 to +0.26)	-0.75 (95% CI -1.33 to -0.17)	43.41	39.44
hsa-PCMTD1_0001	circPCMTD1(2,3).1	-2.23 (95% CI -3.89 to -0.57)	0.033	↑ ARDS	-0.87 (95% CI -3.74 to +2.00)	-2.91 (95% CI -4.94 to -0.88)	2.66	2.57
hsa-EP300_0003	circEP300(7,8,9).1	-1.63 (95% CI -2.86 to -0.39)	0.038	↑ ARDS	-0.68 (95% CI -2.67 to +1.31)	-2.22 (95% CI -3.80 to -0.65)	3.63	4.45
hsa-ASH2L_0010	circASH2L(6,7,L8,9).1	-1.49 (95% CI -2.63 to -0.35)	0.039	↑ ARDS	-1.27 (95% CI -3.47 to +0.92)	-1.57 (95% CI -2.89 to -0.24)	4.02	2.94
hsa-MARCH6_0047	circMARCHF6(21,22).1	+1.15 (95% CI +0.27 to +2.03)	0.039	↑ COVID (S)	+1.46 (95% CI +0.05 to +2.87)	+0.95 (95% CI -0.17 to +2.07)	8.79	6.23
hsa-CHSY1_0001	circCHSY1(2).1	+0.94 (95% CI +0.21 to +1.67)	0.043	↑ COVID (S)	+0.58 (95% CI -0.70 to +1.86)	+1.11 (95% CI +0.22 to +2.00)	7.76	6.88
hsa-EP300_0036	circEP300(3,4,5,6,7,8,9).1	-2.12 (95% CI -3.78 to -0.47)	0.044	↑ ARDS	-1.40 (95% CI -3.82 to +1.01)	-2.76 (95% CI -5.03 to -0.49)	3.54	2.84
hsa-AURKA_0004	circAURKA(4,5,6).1	-1.59 (95% CI -2.84 to -0.33)	0.047	↑ ARDS	-0.58 (95% CI -2.91 to +1.75)	-2.00 (95% CI -3.49 to -0.51)	3.59	3.49
hsa-ANKRD13C_0027	circANKRD13C(4,5S,6,7S,8S,9).1	-1.04 (95% CI -1.86 to -0.22)	0.047	↑ ARDS	-0.20 (95% CI -1.52 to +1.12)	-1.57 (95% CI -2.62 to -0.52)	5.33	6.72
hsa-RAB3D_0003	circRAB3D(3,4).1	+1.15 (95% CI +0.23 to +2.08)	0.052	↑ COVID (S)	+0.34 (95% CI -0.89 to +1.57)	+2.22 (95% CI +0.81 to +3.63)	8.36	7.53

hsa-CCDC66_0010	circCCDC66(8,9,10).1	-1.17 (95% CI -2.11 to -0.22)	0.053	↑ ARDS	-1.32 (95% CI -3.04 to +0.41)	-1.10 (95% CI -2.23 to +0.02)	5.81	6.23
hsa-RP11-407N17_0004	circMIA2(7,8,9).1	+0.76 (95% CI +0.14 to +1.39)	0.058	↑ COVID (S)	+0.79 (95% CI -0.23 to +1.80)	+0.75 (95% CI -0.04 to +1.54)	8.52	9.65
hsa-CPSF6_0003	circCPSF6(7,8,9).1	-0.99 (95% CI -1.80 to -0.17)	0.058	↑ ARDS	-1.31 (95% CI -2.74 to +0.11)	-0.83 (95% CI -1.82 to +0.16)	10.82	11.77
hsa-ASAP1_0002	circASAP1(8,9,10,11,12,13).1	-1.39 (95% CI -2.54 to -0.24)	0.060	↑ ARDS	-1.28 (95% CI -3.02 to +0.45)	-1.47 (95% CI -3.00 to +0.06)	8.25	8.45
hsa-ILKAP_0001	circILKAP(6,7,8,9).1	-1.17 (95% CI -2.18 to -0.16)	0.078	↑ ARDS	-0.06 (95% CI -2.23 to +2.10)	-1.47 (95% CI -2.62 to -0.33)	4.66	5.79
hsa-CCDC66_0011	circCCDC66(RI,5,6).1	-1.01 (95% CI -1.89 to -0.13)	0.082	↑ ARDS	-0.76 (95% CI -2.33 to +0.81)	-1.12 (95% CI -2.18 to -0.05)	5.50	4.37
hsa-DOPEY2_0008	circDOP1B(20,21).1	+1.33 (95% CI +0.16 to +2.50)	0.084	↑ COVID (S)	+0.40 (95% CI -1.61 to +2.41)	+1.80 (95% CI +0.36 to +3.24)	5.27	5.87
hsa-RARS_0012	circRARS1(2,3,4,5).1	-0.69 (95% CI -1.30 to -0.08)	0.084	↑ ARDS	+0.14 (95% CI -1.02 to +1.31)	-1.00 (95% CI -1.71 to -0.29)	10.83	11.13
hsa-SLC43A1_0001	circSLC43A1(9,10,11).1	-1.22 (95% CI -2.31 to -0.13)	0.087	↑ ARDS	-1.10 (95% CI -2.53 to +0.33)	-1.38 (95% CI -3.06 to +0.30)	6.07	3.54
hsa-SLTM_0001	circSLTM(L3,L4,5).1	-0.54 (95% CI -1.01 to -0.06)	0.087	↑ ARDS	-0.40 (95% CI -1.32 to +0.51)	-0.59 (95% CI -1.14 to -0.03)	31.71	34.26
hsa-MAN1A2_0008	circMAN1A2(2,3,4,5,6).1	-0.68 (95% CI -1.29 to -0.07)	0.087	↑ ARDS	+0.19 (95% CI -0.83 to +1.21)	-1.16 (95% CI -1.92 to -0.41)	16.20	13.96
hsa-BPTF_0045	circBPTF(21S,22,23,25,26,27).1	-1.17 (95% CI -2.23 to -0.12)	0.088	↑ ARDS	-1.44 (95% CI -2.93 to +0.05)	-0.91 (95% CI -2.40 to +0.58)	7.83	8.32
hsa-FKBP8_0001	circFKBP8(L5,6).2	-0.62 (95% CI -1.18 to -0.06)	0.092	↑ ARDS	-0.48 (95% CI -1.20 to +0.24)	-0.83 (95% CI -1.73 to +0.06)	23.77	27.38
hsa-CCDC91_0048	circCCDC91(L2,3).2	-0.82 (95% CI -1.56 to -0.07)	0.093	↑ ARDS	-0.80 (95% CI -1.82 to +0.21)	-0.83 (95% CI -1.91 to +0.26)	7.43	6.75

*Combined (Day 3 + Late) effects were estimated by inverse-variance–weighted fixed-effects meta-analysis; two-sided p values were FDR-adjusted (Benjamini–Hochberg). (S)=survival. ARDS= Acute respiratory distress syndrome
