# Supplementary material for: circRNA Signatures Distinguishing COVID-19 Outcomes and Acute Respiratory Distress Syndrome: A Longitudinal, Two-Timepoint, Precision-Weighted Analysis of a Public RNA-Seq Cohort
Source: Genes (Basel). 2025 Dec 30;17(1):34. doi: 10.3390/genes17010034 (PMC12841326; doi:10.3390/genes17010034)
Supplement: Supplementary file 1 [file genes-17-00034-s001.zip › Table S10 Time-Resolved circRNA Differences (Early + Late) - COVID Non-Survivor vs COVID Survivor.pdf]

Table S10:Time-Resolved circRNA Differences (Day 3 + Late) - COVID Non-Survivor vs COVID Survivor

circAtlas ID	Uniform ID	log2FC 95% CI (Combined)*	Adjusted P (Combined)*	Direction	log2FC 95% CI (Early)	log2FC 95% CI (Late)	baseMean (Day3)	baseMean (Late)
hsa-TMCC2_0001	circTMCC2(3).1	+2.67 (95% CI +1.41 to +3.93)	0.01	↑ COVID (NS)	+1.52 (95% CI +0.08 to +2.96)	+6.43 (95% CI +3.82 to +9.04)	20.10	25.48
hsa-ANKRD12_0008	circANKRD12(S8).1	-1.63 (95% CI -2.42 to -0.84)	0.01	↑ COVID (S)	-2.08 (95% CI -3.19 to -0.96)	-1.19 (95% CI -2.30 to -0.08)	27.03	23.06
hsa-UBQLN1_0006	circUBQLN1(2,3,4,5).1	-2.83 (95% CI -4.25 to -1.41)	0.01	↑ COVID (S)	-4.49 (95% CI -6.79 to -2.19)	-1.81 (95% CI -3.62 to -0.01)	4.22	3.30
hsa-VMP1_0001	circVMP1(2,3,4,5).1	-1.38 (95% CI -2.14 to -0.63)	0.02	↑ COVID (S)	-0.75 (95% CI -2.41 to +0.90)	-1.55 (95% CI -2.40 to -0.70)	5.39	8.17
hsa-CLEC16A_0001	circCLEC16A(12,13,14,15,16,17,18,19,20,21,22).1	+1.55 (95% CI +0.68 to +2.42)	0.03	↑ COVID (NS)	+1.51 (95% CI +0.06 to +2.96)	+1.57 (95% CI +0.48 to +2.66)	5.70	8.08
hsa-RANBP9_0002	circRANBP9(6,7,8,9).1	-2.45 (95% CI -3.92 to -0.99)	0.03	↑ COVID (S)	-3.04 (95% CI -5.53 to -0.55)	-2.15 (95% CI -3.96 to -0.33)	3.91	3.02
hsa-ANKRD36BP2	circ(chr2)	+2.26 (95% CI +0.92 to +3.61)	0.03	↑ COVID (NS)	+0.93 (95% CI -1.22 to +3.08)	+3.13 (95% CI +1.40 to +4.86)	6.60	10.80
hsa-CDYL_0005	circCDYL(2).1	+1.16 (95% CI +0.48 to +1.85)	0.03	↑ COVID (NS)	+0.66 (95% CI -0.33 to +1.65)	+1.62 (95% CI +0.67 to +2.57)	48.09	60.55
hsa-RAB6A_0007	circRAB6A(4,6).1	-2.42 (95% CI -3.89 to -0.95)	0.04	↑ COVID (S)	-1.59 (95% CI -4.19 to +1.01)	-2.81 (95% CI -4.59 to -1.03)	3.20	2.48
hsa-KLHL8_0015	circKLHL8(2).1	-1.08 (95% CI -1.77 to -0.40)	0.05	↑ COVID (S)	-1.11 (95% CI -2.15 to -0.07)	-1.06 (95% CI -1.98 to -0.15)	11.04	11.56
hsa-EPB41_0021	circEPB41(10,11).1	+1.93 (95% CI +0.61 to +3.24)	0.09	↑ COVID (NS)	+1.60 (95% CI -0.66 to +3.86)	+2.09 (95% CI +0.48 to +3.70)	4.15	3.99
hsa-SLC14A1_0001	circSLC14A1(7,L8).1	+2.22 (95% CI +0.68 to +3.76)	0.09	↑ COVID (NS)	+0.31 (95% CI -1.84 to +2.46)	+4.24 (95% CI +2.03 to +6.44)	6.35	7.94
hsa-ABHD2_0002	circABHD2(2,3).1	-0.79 (95% CI -1.34 to -0.25)	0.09	↑ COVID (S)	-1.25 (95% CI -2.21 to -0.29)	-0.58 (95% CI -1.24 to +0.09)	12.61	17.52

*Combined (Day 3 + Late) effects were estimated by inverse-variance–weighted fixed-effects meta-analysis; two-sided p values were FDR-adjusted (Benjamini–Hochberg). (NS)=non-survival. (S)=survival
