# Supplementary material for: circRNA Signatures Distinguishing COVID-19 Outcomes and Acute Respiratory Distress Syndrome: A Longitudinal, Two-Timepoint, Precision-Weighted Analysis of a Public RNA-Seq Cohort
Source: Genes (Basel). 2025 Dec 30;17(1):34. doi: 10.3390/genes17010034 (PMC12841326; doi:10.3390/genes17010034)
Supplement: Supplementary file 1 [file genes-17-00034-s001.zip › Table S1a b Summary Statistics for circRNAs detected per group per day.pdf]

Table S1a: Summary Statistics for circRNAs detected per group per day

Day	Group	Number of samples	Mean circRNAs count (≥ 2 reads)	SD	Min circRNAs count	Max circRNAs count
Early	ARDS	4	2058.25	492.34	1549	2494
	COVID (NS)	5	1093.2	268.9	781	1456
	COVID (S)	4	2152.75	1007.63	1127	3072
Late	ARDS	8	2301.38	894.16	1013	3603
	COVID (NS)	9	1227.11	524.03	621	2441
	COVID (S)	6	1391	455.39	633	1962
Pairwise Wilcoxon among the groups						
Day	**Disease status**	**Adjusted p-value**				
Early	ARDS vs COVID (NS)	0.06				
	ARDS vs COVID (S)	1				
	COVID (NS) vs COVID (S)	0.167				
Late	ARDS vs COVID (NS)	0.04				
	ARDS vs COVID (S)	0.122				
	COVID (NS) vs COVID (S)	0.263				

ARDS = Acute respiratory distress syndrome, COVID (NS) = COVID non-survival, COVID (S) = COVID survival.

Table S1b: Study design details: individuals per group per timepoint, Early–Late pairing, and repeated-measure sampling

Run ID	Disease status	Subject ID	Time
SRR29999123	COVID (S)	S1	Day 10
SRR29999124	COVID (S)		Day 7
SRR29999125	COVID (S)		Day 3
SRR29999120	COVID (S)	S2	Day 7
SRR29999121	COVID (S)		Day 3
SRR29999116	COVID (S)	S3	Day 10
SRR29999117	COVID (S)		Day 7
SRR29999118	COVID (S)		Day 3
SRR29999113	COVID (S)	S4	Day 7
SRR29999114	COVID (S)		Day 3
SRR29999109	COVID (NS)	NS1	Day 10
SRR29999110	COVID (NS)		Day 7
SRR29999111	COVID (NS)		Day 3
SRR29999105	COVID (NS)	NS2	Day 10
SRR29999106	COVID (NS)		Day 7
SRR29999107	COVID (NS)		Day 3
SRR29999101	COVID (NS)	NS3	Day 10
SRR29999102	COVID (NS)		Day 7
SRR29999103	COVID (NS)		Day 3
SRR29999098	COVID (NS)	NS4	Day 7
SRR29999099	COVID (NS)		Day 3
SRR29999095	COVID (NS)	NS5	Day 7
SRR29999096	COVID (NS)		Day 3
SRR29999127	COVID (NS)		Day 10
SRR29999140	ARDS	C1	Day 10
SRR29999141	ARDS		Day 7
SRR29999142	ARDS		Day 3
SRR29999136	ARDS	C2	Day 10
SRR29999137	ARDS		Day 7

SRR29999138	ARDS		Day 3
SRR29999132	ARDS	C3	Day 10
SRR29999133	ARDS		Day 7
SRR29999134	ARDS		Day 3
SRR29999128	ARDS	C4	Day 10
SRR29999129	ARDS		Day 7
SRR29999130	ARDS		Day 3
