# Supplementary material for: circRNA Signatures Distinguishing COVID-19 Outcomes and Acute Respiratory Distress Syndrome: A Longitudinal, Two-Timepoint, Precision-Weighted Analysis of a Public RNA-Seq Cohort
Source: Genes (Basel). 2025 Dec 30;17(1):34. doi: 10.3390/genes17010034 (PMC12841326; doi:10.3390/genes17010034)
Supplement: Supplementary file 1 [file genes-17-00034-s001.zip › Table S1c Sequencing depth and STAR alignment summary per sample.pdf]

Table S1c: Sequencing depth and STAR alignment summary per sample

RunID	Input reads	Uniquely mapped reads	Uniquely mapped percent
SRR29999095	39808906	33948807	85.28%
SRR29999096	39236156	34299327	87.42%
SRR29999097	37232966	33036061	88.73%
SRR29999098	35925427	32063531	89.25%
SRR29999099	36202963	30943046	85.47%
SRR29999100	35482783	30232737	85.20%
SRR29999101	37419110	33721618	90.12%
SRR29999102	39566715	34312196	86.72%
SRR29999103	40007697	34292005	85.71%
SRR29999104	40772015	32835515	80.53%
SRR29999105	42731821	29975262	70.15%
SRR29999106	39880993	32590564	81.72%
SRR29999107	45404172	37406075	82.38%
SRR29999108	42011360	37591724	89.48%
SRR29999109	44003019	35033386	79.62%
SRR29999110	44257435	33577623	75.87%
SRR29999111	38934528	28945544	74.34%
SRR29999112	37615058	31791609	84.52%
SRR29999113	39431146	36668715	92.99%
SRR29999114	45850488	40415563	88.15%
SRR29999115	51858407	38411308	74.07%
SRR29999116	46179026	41637417	90.17%
SRR29999117	53832804	45472508	84.47%
SRR29999118	46026056	41200201	89.51%
SRR29999119	45730048	41821067	91.45%
SRR29999120	41848936	38014975	90.84%
SRR29999121	44902541	40334767	89.83%
SRR29999122	48487021	43066833	88.82%
SRR29999123	43335518	39563762	91.30%
SRR29999124	44906741	39910534	88.87%
SRR29999125	39867571	34532974	86.62%
SRR29999126	43019268	32336927	75.17%
SRR29999127	42708782	33470478	78.37%
SRR29999128	44622352	35005784	78.45%
SRR29999129	43667940	30005046	68.71%
SRR29999130	38176480	34398648	90.10%
SRR29999131	40400278	36511281	90.37%
SRR29999132	44461055	26886770	60.47%
SRR29999133	48618954	38326984	78.83%
SRR29999134	41954980	35072965	83.60%
SRR29999135	44577511	40096286	89.95%
SRR29999136	45228652	27200728	60.14%
SRR29999137	45199362	31084718	68.77%
SRR29999138	49681737	38606176	77.71%

SRR29999139	35749569	30940085	86.55%
SRR29999140	47453473	39361330	82.95%
SRR29999141	42760293	36717338	85.87%
SRR29999142	41364129	36617519	88.52%
SRR29999143	39932136	36359346	91.05%
