# Supplementary material for: circRNA Signatures Distinguishing COVID-19 Outcomes and Acute Respiratory Distress Syndrome: A Longitudinal, Two-Timepoint, Precision-Weighted Analysis of a Public RNA-Seq Cohort
Source: Genes (Basel). 2025 Dec 30;17(1):34. doi: 10.3390/genes17010034 (PMC12841326; doi:10.3390/genes17010034)
Supplement: Supplementary file 1 [file genes-17-00034-s001.zip › Table S2 Top differentially expressed circRNAs between COVID non-survival and ARDS at early (Day 3) stage.pdf]

Table S2: Top differentially expressed circRNAs between COVID non-survival and ARDS at early (Day 3) stage

circAtlas ID	Uniform ID	Gene name	baseMean	Log2Fold Change	lfcSE	Stat	pvalue	padj
hsa-ANKRD12_0008	circANKRD12(S8).1	ANKRD12	27.03	-2.81	0.56	-4.98	0.00000065	0.0002
hsa-UBQLN1_0006	circUBQLN1(2,3,4,5).1	UBQLN1	4.22	-4.96	1.17	-4.25	0.00002155	0.0037
hsa-TMEM165_0001	circTMEM165(2,3,4).1	TMEM165	4.67	-4.96	1.24	-4.01	0.00006174	0.0071
hsa-ITGAL_0003	circITGAL(RI,14,15).1	ITGAL	15.00	2.00	0.52	3.86	0.000113666	0.0098
hsa-FAM13B_0019	circFAM13B(8,9,10).1	FAM13B	18.51	-2.10	0.59	-3.53	0.000420942	0.0291
hsa-IL27RA_0001	circIL27RA(5,6).1	IL27RA	7.53	2.51	0.73	3.46	0.00053391	0.0295
hsa-PCMTD1_0002	circPCMTD1(2).1	PCMTD1	23.33	-1.82	0.53	-3.43	0.000595977	0.0295
hsa-METTL3_0002	circMETTL3(2,2,RI,3).1	METTL3	12.19	1.64	0.50	3.27	0.001068067	0.0348
hsa-DHX34_0001	circDHX34(6).1	DHX34	6.15	2.64	0.82	3.23	0.001255756	0.0348
hsa-MAN1A2_0003	circMAN1A2(2,3,4,5).1	MAN1A2	26.48	-1.57	0.49	-3.21	0.001307866	0.0348
hsa-UXS1_0001	circUXS1(2,3S,4,5).1	UXS1	5.88	2.72	0.84	3.24	0.001210854	0.0348
hsa-XPO1_0001	circXPO1(2,3,4).1	XPO1	19.33	-2.06	0.64	-3.22	0.001300439	0.0348
hsa-MARCH6_0047	circMARCHF6(21,22).1	MARCHF6	8.79	2.30	0.69	3.33	0.000869438	0.0348
hsa-DEF6_0002	circDEF6(RI,4,5).1	DEF6	9.69	2.12	0.67	3.17	0.001527256	0.0352
hsa-GAPVD1_0019	circGAPVD1(16,17).1	GAPVD1	3.63	-3.69	1.16	-3.17	0.001508981	0.0352
hsa-ZNF516_0005	circZNF516(S3).1	ZNF516	11.26	2.03	0.65	3.14	0.001697709	0.0367
hsa-XPO6_0027	circXPO6(5,6).1	XPO6	6.87	2.20	0.73	3.02	0.002538516	0.0446
hsa-RSRC1_0001	circRSRC1(2,3).1	RSRC1	4.67	-2.65	0.88	-3.01	0.002578945	0.0446
hsa-MAN2A1_0001	circMAN2A1(2,3,4).1	MAN2A1	6.38	-2.62	0.86	-3.05	0.002299761	0.0446
circFOXO3	circFOXO3	FOXO3	4.50	-4.23	1.40	-3.03	0.002464863	0.0446
hsa-RANBP9_0002	circRANBP9(6,7,8,9).1	RANBP9	3.91	-3.73	1.26	-2.97	0.003000687	0.0494
hsa-PPFIA1_0003	circPPFIA1(17,18,19).1	PPFIA1	4.81	-2.27	0.77	-2.94	0.003298024	0.0500
hsa-ERBIN_0001	circERBIN(2,3,4).1	ERBIN	2.71	-3.91	1.33	-2.94	0.003325801	0.0500
hsa-PICALM_0008	circPICALM(2,3,4,5,6,7).1	PICALM	3.70	-3.86	1.32	-2.92	0.003478281	0.0501
hsa-ASAP1_0002	circASAP1(8,9,10,11,12,13).1	ASAP1	8.25	-2.71	0.94	-2.88	0.00393387	0.0544
hsa-TBCEL_0004	circTBCEL(3,4,5S,6,7S,8).1	TBCEL	4.97	-2.23	0.79	-2.84	0.004514055	0.0601
hsa-AXIN1_0011	circAXIN1(3,4,5).1	AXIN1	8.77	1.87	0.67	2.81	0.004927804	0.0631
hsa-NFATC3_0001	circNFATC3(2,3).1	NFATC3	12.55	-1.16	0.42	-2.79	0.005252709	0.0649
hsa-GCN1_0003	circGCN1(29,30,31).1	GCN1	10.78	2.16	0.78	2.78	0.005486841	0.0655
hsa-PICALM_0001	circPICALM(2,3,4,5,6,7,8,9,10,11,12S).1	PICALM	7.16	-2.74	1.00	-2.74	0.006074	0.0663
hsa-MYO9B_0005	circMYO9B(2).1	MYO9B	14.59	1.99	0.72	2.76	0.005837036	0.0663
hsa-VRK2_0001	circVRK2(3,4,5,6,7).1	VRK2	3.80	-3.50	1.28	-2.74	0.006130195	0.0663

hsa-SPECC1_0001	circSPECC1(4).1	SPECC1	89.11	-1.90	0.70	-2.71	0.006712303	0.0704
hsa-ITGAL_0005	circITGAL(5,6).1	ITGAL	6.58	1.97	0.75	2.62	0.008921595	0.0908

baseMean: Average expression level across all samples. log2FoldChange: Log2-transformed fold change between two conditions, Negative value means downregulated in COVID non-survival and positive means upregulated in COVID non-survival . lfcSE: log2 fold change of standard error. Stat: Statistical test value for differential expression. pvalue: Raw p-value from the statistical test. padj: Adjusted p-value (corrected for multiple testing).

Based on the ≥2 BSJ count matrix, included for transparency. Primary conclusions rely on the two-time-point, precision-weighted Early–Late analysis
