# Supplementary material for: circRNA Signatures Distinguishing COVID-19 Outcomes and Acute Respiratory Distress Syndrome: A Longitudinal, Two-Timepoint, Precision-Weighted Analysis of a Public RNA-Seq Cohort
Source: Genes (Basel). 2025 Dec 30;17(1):34. doi: 10.3390/genes17010034 (PMC12841326; doi:10.3390/genes17010034)
Supplement: Supplementary file 1 [file genes-17-00034-s001.zip › Table S3 Top differentially expressed circRNAs between COVID non-survival and ARDS at late (Day 7+10) stage.pdf]

Table S3: Top differentially expressed circRNAs between COVID non-survival and ARDS at late (Day 7+10) stage

circAtlas ID	Uniform ID	Gene name	baseMean	Log2Fold Change	lfcSE	Stat	pvalue	padj
hsa-TFRC_0013	circTFRC(3,4).1	TFRC	4.18	-2.75	0.61	-4.53	0.00001	0.002
hsa-PCMTD1_0002	circPCMTD1(2).1	PCMTD1	26.84	-1.81	0.44	-4.14	0.00003	0.005
hsa-NRIP1_0002	circNRIP1(2,3).1	NRIP1	5.77	2.57	0.68	3.81	0.00014	0.013
hsa-MINPP1_0001	circMINPP1(2,3,L4).1	MINPP1	2.40	-3.50	0.94	-3.72	0.00020	0.015
hsa-DNAJC6_0001	circDNAJC6(2,3,4).1	DNAJC6	9.90	-2.30	0.64	-3.58	0.00034	0.016
hsa-PCNT_0003	circPCNT(7,8).1	PCNT	7.58	-2.20	0.61	-3.62	0.00030	0.016
hsa-RAB6A_0007	circRAB6A(4,6).1	RAB6A	2.48	-3.00	0.85	-3.52	0.00043	0.017
hsa-RNF10_0004	circRNF10(RI,5,6).1	RNF10	15.77	-1.14	0.33	-3.50	0.00046	0.017
hsa-ANKRD12_0008	circANKRD12(S8).1	ANKRD12	23.06	-1.78	0.52	-3.43	0.00061	0.019
hsa-RBM33_0009	circRBM33(3,4,5).1	RBM33	22.08	-1.10	0.33	-3.39	0.00069	0.020
hsa-SLC45A4_0002	circSLC45A4(2).1	SLC45A4	13.45	0.93	0.28	3.36	0.00079	0.021
hsa-RNF10_0006	circRNF10(5,6,7).1	RNF10	3.15	-2.41	0.74	-3.24	0.00118	0.026
hsa-CREBBP_0001	circCREBBP(2).1	CREBBP	8.08	-1.03	0.32	-3.25	0.00115	0.026
hsa-SLTM_0001	circSLTM(L3,L4,5).1	SLTM	34.26	-0.83	0.26	-3.17	0.00150	0.031
hsa-PICALM_0007	circPICALM(2,3,4,5,6,L7,8S).1	PICALM	4.24	-2.12	0.69	-3.09	0.00203	0.036
hsa-FBXO9_0002	circFBXO9(L4).1	FBXO9	5.26	-1.43	0.46	-3.10	0.00194	0.036
hsa-ASH2L_0010	circASH2L(6,7,L8,9).1	ASH2L	2.94	-2.00	0.65	-3.08	0.00210	0.036
hsa-UBQLN1_0006	circUBQLN1(2,3,4,5).1	UBQLN1	3.30	-2.57	0.84	-3.06	0.00221	0.036
hsa-GMIP_0001	circGMIP(5,RI,6,7).1	GMIP	11.07	1.11	0.38	2.95	0.00314	0.048
hsa-TMEM56-RWDD3_0004	circTLCD4(2S,3,L4,5).1	TLCD4	19.36	-2.08	0.72	-2.87	0.00405	0.056
hsa-VRK2_0001	circVRK2(3,4,5,6,7).1	VRK2	3.13	-2.17	0.75	-2.88	0.00401	0.056
hsa-FCHO2_0038	circFCHO2(20,21).1	FCHO2	8.42	-1.33	0.47	-2.86	0.00422	0.056
hsa-ANKRD36BP2	circ(chr2)	ANKRD36BP2	10.80	-1.61	0.57	-2.81	0.00502	0.061
hsa-CCDC66_0011	circCCDC66(RI,5,6).1	CCDC66	4.37	-1.44	0.51	-2.81	0.00490	0.061
hsa-FCHO2_0068	circFCHO2(17,18,19S,20,L21).1	FCHO2	5.12	-1.90	0.69	-2.77	0.00559	0.065
hsa-COPA_0003	circCOPA(6,7,8).1	COPA	5.36	-1.25	0.45	-2.75	0.00591	0.065
hsa-AKAP7_0001	circAKAP7(2,L3,4,5).1	AKAP7	5.16	-1.76	0.64	-2.74	0.00609	0.065
hsa-SUCO_0003	circSUCO(2,3,L4,5,6).1	SUCO	6.63	-1.83	0.67	-2.72	0.00660	0.066
hsa-RHBDD1_0004	circRHBDD1(4,5).1	RHBDD1	12.11	-1.37	0.50	-2.73	0.00640	0.066
hsa-TBCEL_0004	circTBCEL(3,4,5S,6,7S,8).1	TBCEL	5.33	-1.25	0.47	-2.67	0.00766	0.068
hsa-SCARF1_0001	circSCARF1(9,RI,10).1	SCARF1	6.38	1.53	0.57	2.67	0.00764	0.068
hsa-ZNF516_0005	circZNF516(S3).1	ZNF516	7.78	1.48	0.56	2.67	0.00764	0.068

hsa-ASAP1_0002	circASAP1(8,9,10,11,12,13).1	ASAP1	8.45	-1.91	0.72	-2.66	0.00772	0.068
hsa-CCT2_0003	circCCT2(7,8,9,L10).1	CCT2	4.00	-1.74	0.67	-2.61	0.00916	0.070
hsa-HERC1_0013	circHERC1(22,23,24L,25).1	HERC1	2.83	-1.85	0.71	-2.62	0.00877	0.070
hsa-PHLPP2_0024	circPHLPP2(L6,7,8).1	PHLPP2	2.04	-1.89	0.72	-2.61	0.00899	0.070
hsa-FECH_0013	circFECH(L2,3,4).1	FECH	2.50	-1.64	0.63	-2.62	0.00885	0.070
hsa-ARHGAP26_0002	circARHGAP26(15,16,17).1	ARHGAP26	7.48	1.12	0.43	2.63	0.00859	0.070
hsa-PICALM_0008	circPICALM(2,3,4,5,6,7).1	PICALM	3.96	-1.77	0.69	-2.56	0.01059	0.077
hsa-UBR2_0001	circUBR2(5,6,7).1	UBR2	2.44	-2.18	0.86	-2.55	0.01090	0.077
hsa-EZH2_0001	circEZH2(2,3).1	EZH2	3.20	-1.66	0.65	-2.55	0.01070	0.077
hsa-ZMYND8_0005	circZMYND8(8,L9,10,11).1	ZMYND8	5.37	-1.11	0.44	-2.51	0.01214	0.084
hsa-SUCO_0022	circSUCO(4,5,6).1	SUCO	3.37	-1.89	0.76	-2.49	0.01279	0.084
hsa-ZCCHC6_0015	circTUT7(19,20,21,22,23).1	TUT7	4.67	-1.53	0.61	-2.50	0.01251	0.084
hsa-XPO1_0001	circXPO1(2,3,4).1	XPO1	18.27	-1.10	0.44	-2.47	0.01342	0.086
hsa-RCL1_0008	circRCL1(2,3).1	RCL1	5.33	-1.67	0.68	-2.46	0.01382	0.087
hsa-SCNM1_0001	circSCNM1(3,RI,4,5).1	SCNM1	6.60	-1.19	0.49	-2.42	0.01533	0.095
hsa-RHBDD1_0003	circRHBDD1(4,5,6,7,8).1	RHBDD1	10.59	-1.35	0.56	-2.41	0.01594	0.096
hsa-MBOAT2_0001	circMBOAT2(2,3).1	MBOAT2	3.97	-1.88	0.78	-2.40	0.01642	0.097

baseMean: Average expression level across all samples. log2FoldChange: Log2-transformed fold change between two conditions, Negative value means downregulated in COVID non-survival and positive means upregulated in COVID non-survival . lfcSE: log2 fold change of standard error. Stat: Statistical test value for differential expression. pvalue: Raw p-value from the statistical test. padj: Adjusted p-value (corrected for multiple testing).

Based on the ≥2 BSJ count matrix, included for transparency. Primary conclusions rely on the two-time-point, precision-weighted Early–Late analysis
