# Supplementary figures and images for: circRNA Signatures Distinguishing COVID-19 Outcomes and Acute Respiratory Distress Syndrome: A Longitudinal, Two-Timepoint, Precision-Weighted Analysis of a Public RNA-Seq Cohort
Source: Genes (Basel). 2025 Dec 30;17(1):34. doi: 10.3390/genes17010034 (PMC12841326; doi:10.3390/genes17010034)

# Volcano Plot: circRNA differential expression

COVID (NS) vs ARDS

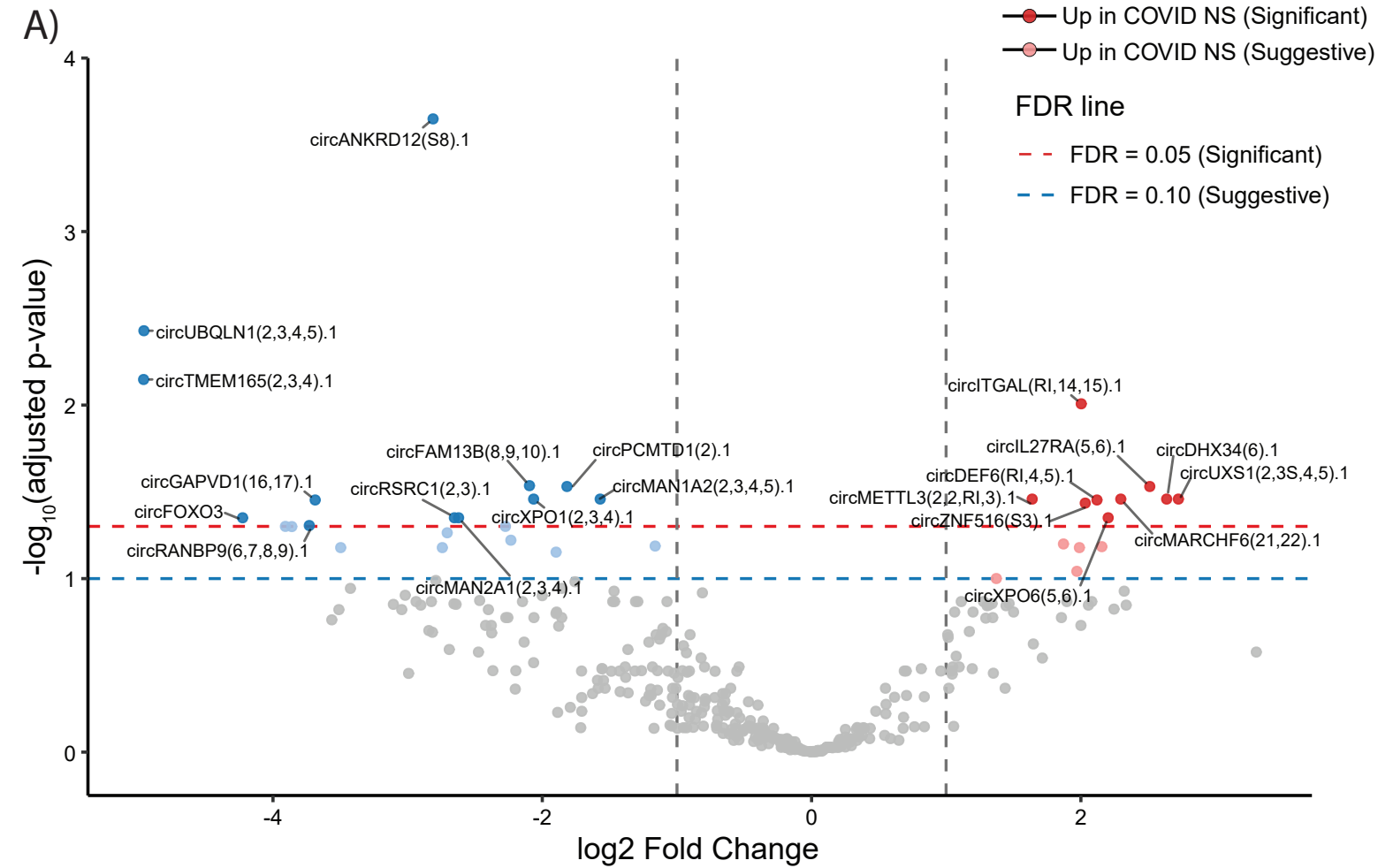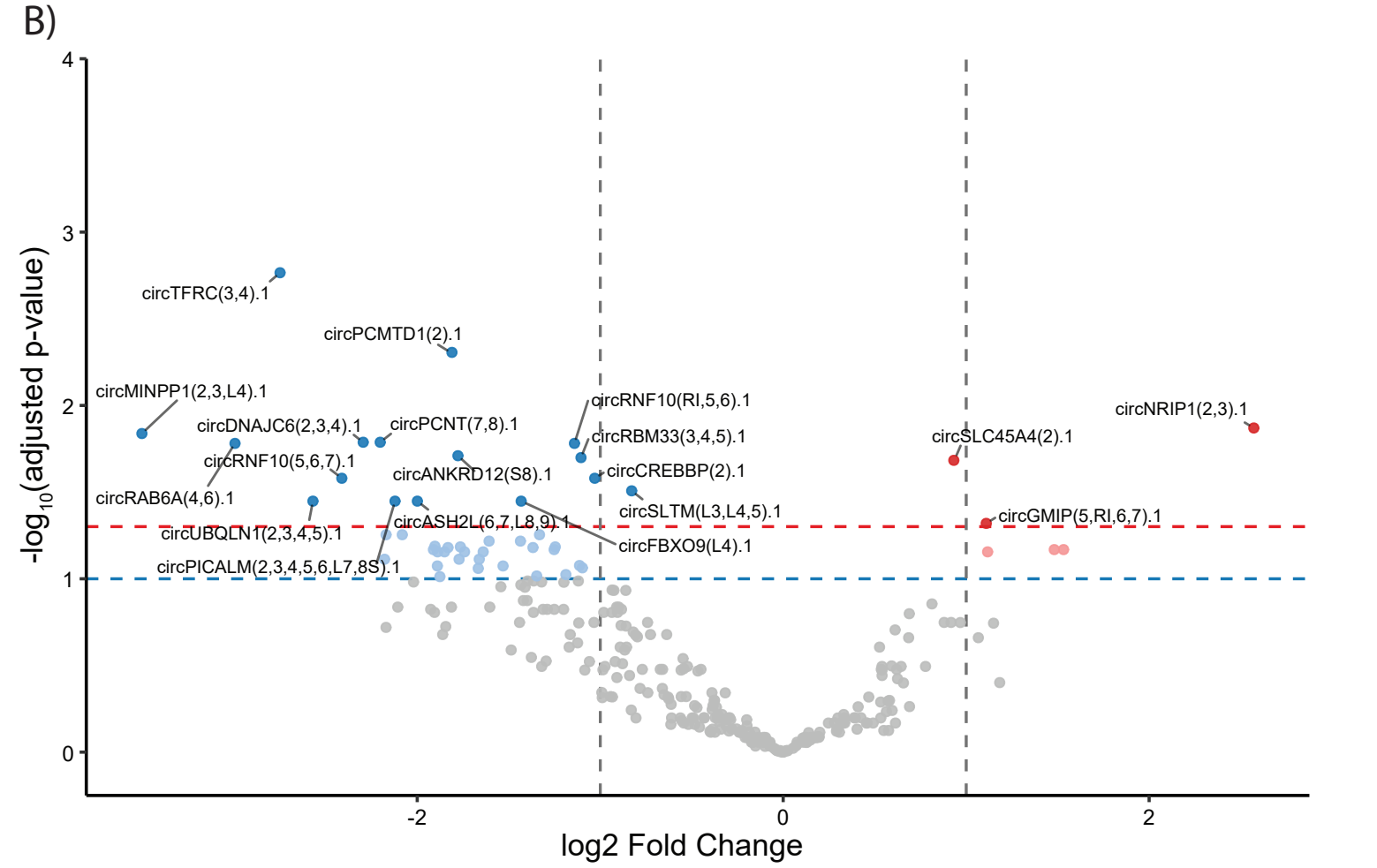

Supplement: Supplementary file 1 [file genes-17-00034-s001.zip › Figure S1 Volcano plot circRNA differential expression COVID (NS) vs ARDS early and late.pdf]

Volcano Plot: circRNA differential expression

COVID (S) vs ARDS

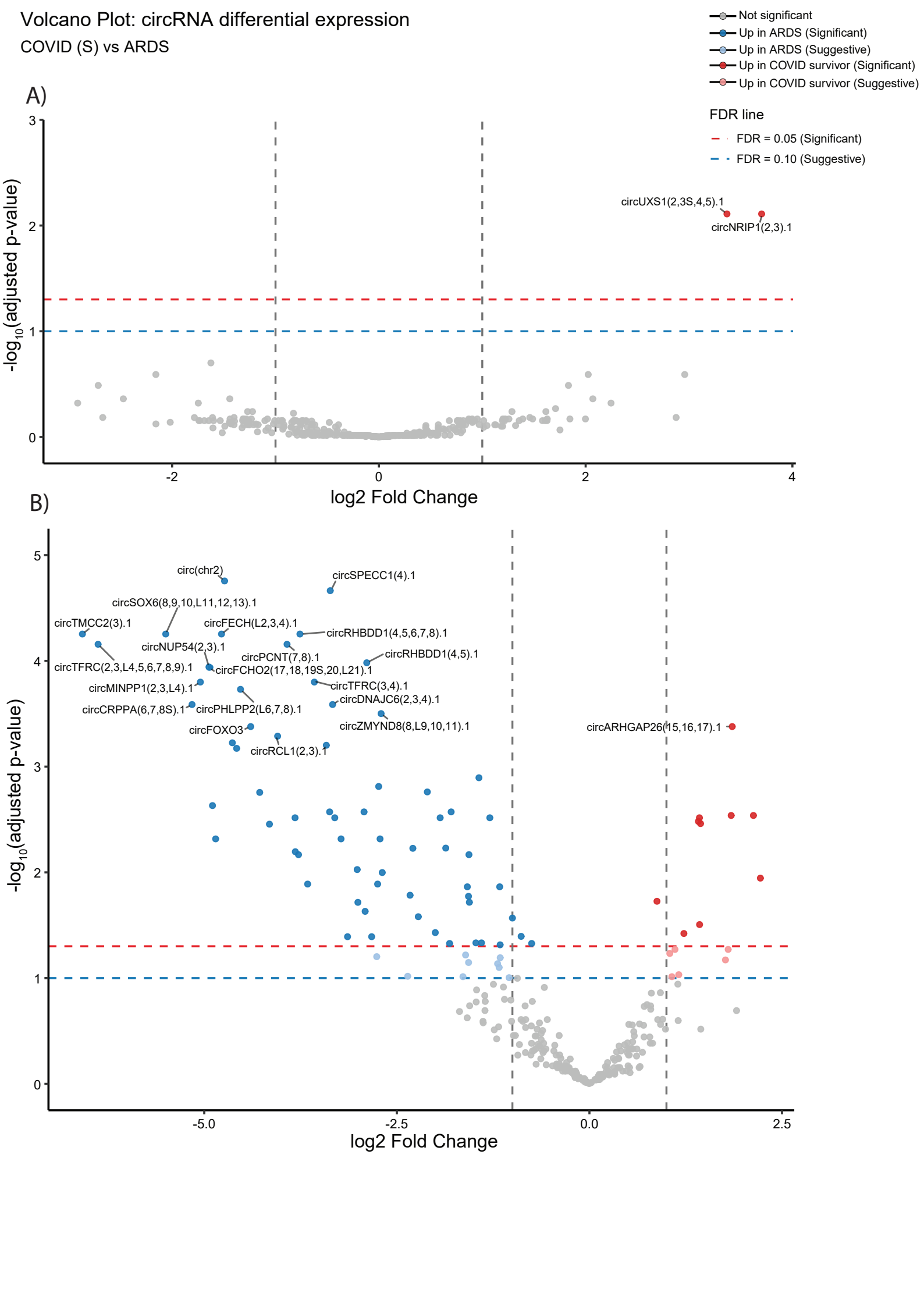

Supplement: Supplementary file 1 [file genes-17-00034-s001.zip › Figure S2 Volcano plot circRNA differential expression COVID (S) vs ARDS early and late.pdf]

# Volcano Plot: circRNA differential expression

COVID (NS) vs COVID (S)

A)

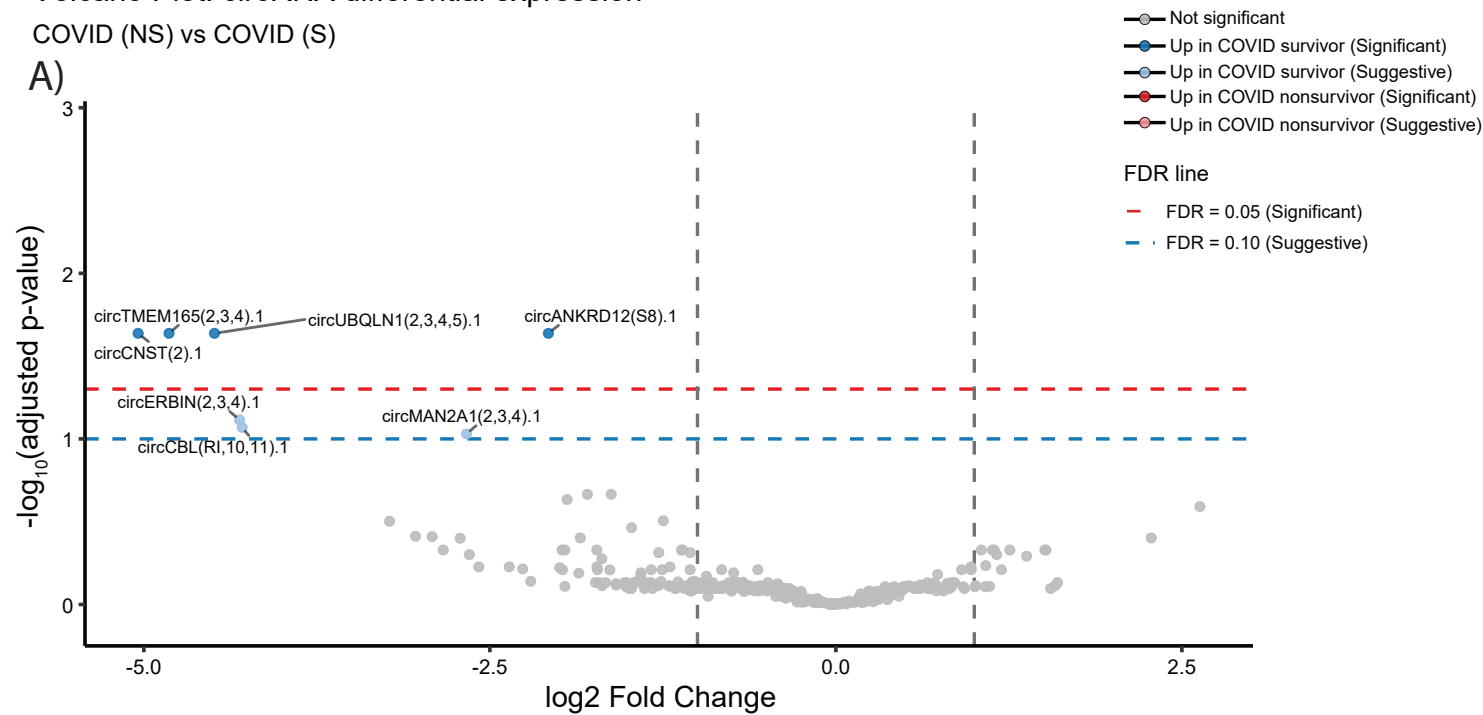

B)

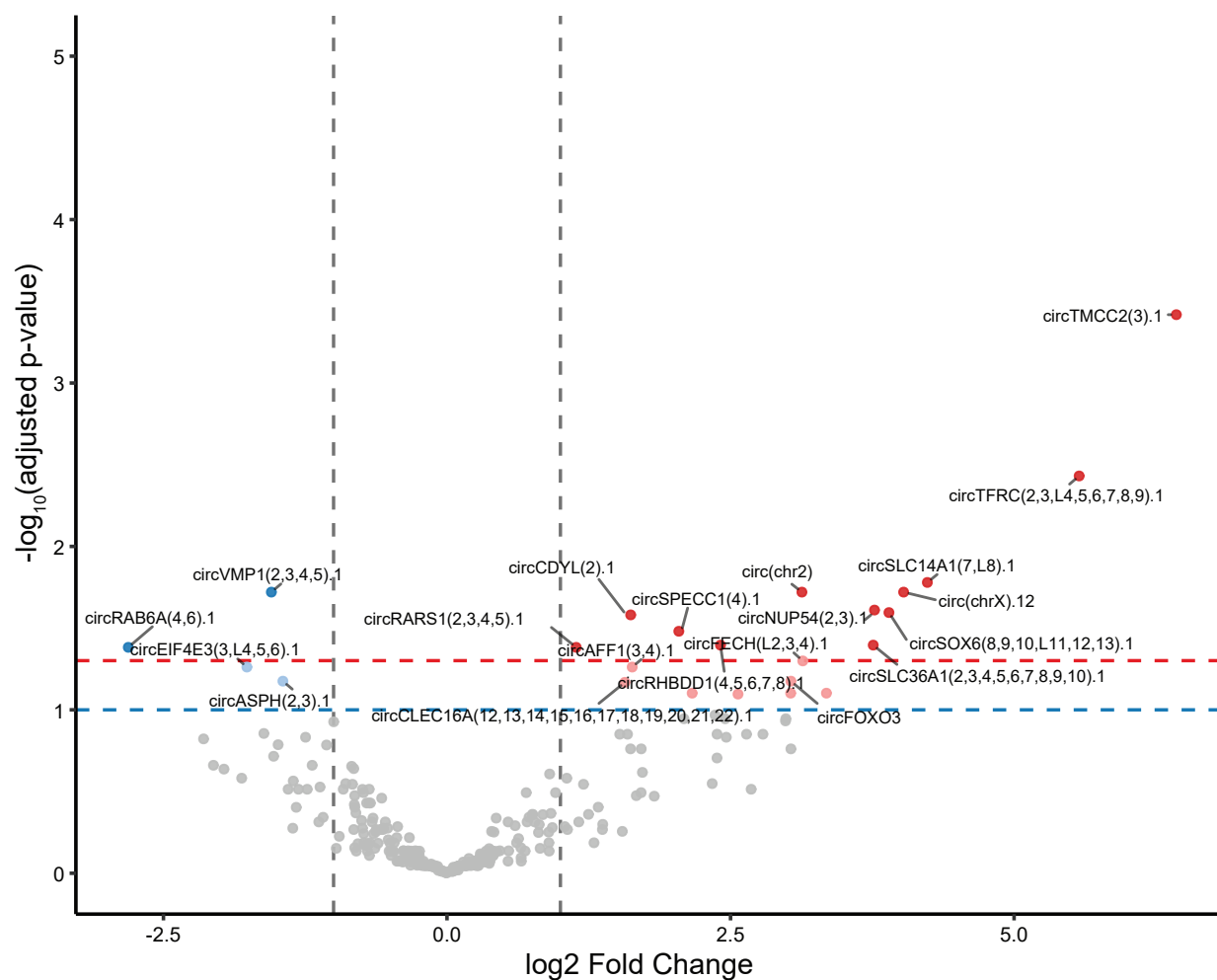

Supplement: Supplementary file 1 [file genes-17-00034-s001.zip › Figure S3 Volcano plot circRNA differential expression COVID (NS) vs COVID (S) early and late.pdf]
